# Bayesian identification of structural coefficients in causal models and the causal false-positive risk of confounders and colliders in linear Markovian models

**DOI:** 10.1186/s12874-021-01473-w

**Published:** 2022-02-27

**Authors:** Riko Kelter

**Affiliations:** Department of Mathematics, University of Siegen, Walter-Flex-Str. 3, Siegen, Germany

**Keywords:** Causal inference, Bayesian inference, Directed acyclic graph (DAG), d-separation, Bayes factor, Structural coefficients

## Abstract

**Background:**

Causal inference has seen an increasing popularity in medical research. Estimation of causal effects from observational data allows to draw conclusions from data when randomized controlled trials cannot be conducted. Although the identification of structural causal models (SCM) and the calculation of structural coefficients has received much attention, a key requirement for valid causal inference is that conclusions are drawn based on the true data-generating model.

**Methods:**

It remains widely unknown how large the probability is to reject the true structural causal model when observational data from it is sampled. The latter probability – the causal false-positive risk – is crucial, as rejection of the true causal model can induce bias in the estimation of causal effects. In this paper, the widely used causal models of confounders and colliders are studied regarding their causal false-positive risk in linear Markovian models. A simulation study is carried out which investigates the causal false-positive risk in Gaussian linear Markovian models. Therefore, the testable implications of the DAG corresponding to confounders and colliders are analyzed from a Bayesian perspective. Furthermore, the induced bias in estimating the structural coefficients and causal effects is studied.

**Results:**

Results show that the false-positive risk of rejecting a true SCM of even simple building blocks like confounders and colliders is substantial. Importantly, estimation of average, direct and indirect causal effects can become strongly biased if a true model is rejected. The causal false-positive risk may thus serve as an indicator or proxy for the induced bias.

**Conclusion:**

While the identification of structural coefficients and testable implications of causal models have been studied rigorously in the literature, this paper shows that causal inference also must develop new concepts for controlling the causal false-positive risk. Although a high risk cannot be equated with a substantial bias, it is indicative of the induced bias. The latter fact calls for the development of more advanced risk measures for committing a causal type I error in causal inference.

**Supplementary Information:**

The online version contains supplementary material available at (10.1186/s12874-021-01473-w).

## Background

**Fig. 1:**
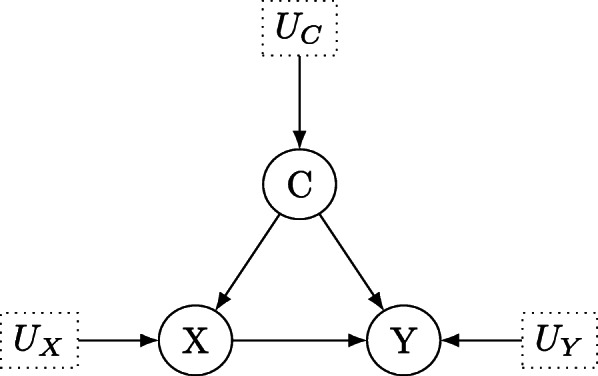
Directed acyclic graph for the structural causal model which describes the effect of treatment *X* on outcome *Y* under the confounding variable *C*. *U*_*X*_, *U*_*Y*_ and *U*_*C*_ denote unobservable latent variables, which determine the values of *X*, *Y* and *C*

Causal inference deals with the identification of causes and quantification of causal effects in experimental or purely observational data [1, 2]. While much of statistical science has dealt with developing the mathematical theory for parameter estimation, hypothesis testing or confidence set construction, a variety of scientifically important questions remains unsolved when purely statistical means are taken into account. Policy makers as well as researchers are interested not only in the predictions of a statistical model or parameter estimates of some parameters of interest but in the effect of interventions or policy manipulations. For example, when considering the development of a new drug, a relevant question is whether the drug can be considered as causal for the treatment of the disease, that is, can a positive effect observed between patients taking the drug and patients who do not take it be attributed to the administration of the drug? As is well known correlation is not causation so observing a correlation between improved health condition and taking the new drug *in purely observational data* alone does not suffice to attribute the drug as causal for the effect. The situation is depicted in Fig. 1, where the treatment *X* could resemble the new drug, the outcome *Y* the health condition of an individual, and *C* a possible confounding variable. Such a confounder could be the health condition of an individual: Maybe individuals who are in a generally better health condition and suffer from a weaker form of the disease are more likely to take the drug. These patients will also be more likely to recover, and thus *C* affects both the access to the treatment and the outcome, confounding the causal effect of the drug *X* on outcome *Y*.

Likewise, when a predictive model states that patients will recover earlier after surgery when being assigned to additional physical therapy (PT), a policy maker will typically ask: “Is additional physical therapy causal for the faster recovery of patients after surgery?”. Purely observational data present a major obstacle in answering such a question, simply because it may happen that patients who are in a better health condition after the surgery (for whom the surgery worked better) could show up more frequently at PT. Patients who did not show up that frequently at additional physical therapy could have been in a worse health condition (the surgery was not as effective as for the other patients) and as a consequence physical therapy mediates the true effect of the treatment on recovery time. It could be the case that 90% of the faster recovery can be attributed to the mediator, while the surgery only accounts for 10% of the total causal effect. Attribution of causal effects is thus crucial to provide insights into the effectiveness of such interventions. Causal inference provides means to achieve such insights from purely observational data.

**Fig. 2:**
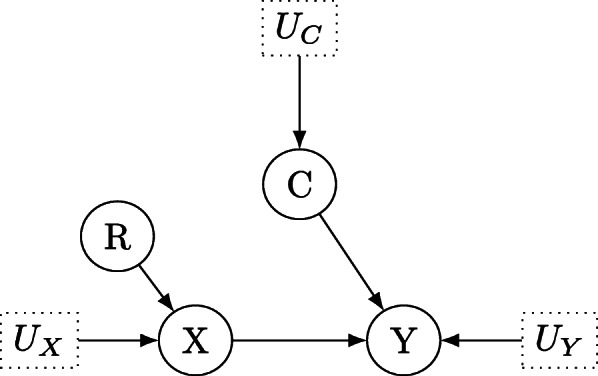
Directed acyclic graph for the structural causal model which describes the effect of treatment *X* on outcome *Y* under randomization *R*

One may wonder why performing a randomized controlled trial (RCT) is not the ultimate solution to the above dilemma. Since the early days of Fisher [3], randomized controlled trials have become the gold standard in biomedical research, which is for good reasons: Randomization ensures that undesired influences can be excluded from the analysis by eliminating confounders and this in turn enables researchers to interpret observed differences as causal. Consider the situation depicted in Fig. 2. Figure 2 shows the same situation as in Fig. 1, but this time a randomization scheme *R* is added to determine who is administered the treatment (or drug) *X*. Now individuals are not free in their choice of opting for the treatment *X* or not, and the influence of confounding variables like the gender, overall health condition, et cetera – each of which could stand for the confounding variable *C* in Fig. 1 – can efficiently be eliminated. This is depicted as the missing arrow *C*→*X* in Fig. 2 and the effect of the treatment *X* on the outcome *Y* and the variable *C* on the outcome *Y* can now clearly be distinguished. This is in sharp contrast to Fig. 1 where it is unclear which fraction of the effect of *X* on *Y* is attributable to *X*, and which fraction is attributable to *C*. A randomized controlled trial helps in this first example, but the influence of the mediator physical therapy in the second example can not so easily be eliminated by randomization: Even after patients have been randomized into the treatment and control groups, the effect of the treatment *X* can be mediated through a mediator *Mafter* the treatment *X* has been applied. As a consequence, mediation analysis is mandatory even in RCTs [4]. In linear systems, a minimal requirement is to separate between the direct and indirect effect of *X* on *Y*, while in non-linear systems the language of counterfactuals is required, see Pearl et al. [2], Section 3.8.4.

An important preliminary for a RCT is that experimental intervention is possible after all. In a variety of cases however, ethical concerns do not allow to perform a RCT. For example, studying the effect of sleep deprivation on cognitive ability by forcing people to stay awake is not possible due to the known adverse effects of sleep deprivation on the general health condition [5]. Also, economic considerations such as the costs associated with performing an RCT present challenges. Even if these challenges are met, RCTs still suffer from aspects such as compliance or loss to follow-up. In contrast, purely observational data can be thought of as somehow ideal in the sense that study participants are observed under their natural living conditions. The transferability of causal inference from observational data to the everyday life of individuals thus seems better than when individuals are being aware that they are participating in a RCT. For example, the latter case could cause a deliberate change in the behaviour of RCT participants (e.g. because of social desirability bias). In sum, while RCTs often provide a meaningful way to obtain causal inferences by eliminating possible confounders, methods for the analysis of non-interventional purely observational data are required to answer questions where performing a RCT is simply not possible due to ethical, economic or conceptual obstacles.

### Contribution

An important condition for the validity of causal inferences from either observational or experimental data is that the underlying structural causal model which is often represented by a directed acyclic graph (DAG) is *correct*. If causal inferences are based on a structural causal model which does *not* represent the true data-generating process, estimates for a causal effect can become biased and lead to unreliable attributions of causal effects to a set of variables.^1^ This stresses that model validation is a key requirement in causal inference from observational or experimental data, and checking the testable implications of the structural causal model is therefore mandatory before any causal effects are calculated. In case one or multiple of the testable implications of the assumed model are violated, the model must be refined before any causal effect is computed or the model must be entirely rejected. Refinement of the model may be possible in some cases, keeping a causal effect computable, while in others the structure of the model must be modified substantially so that the calculation of a causal effect may turn out to require an entirely different method or even become impossible. In the latter case, the effect is not identifiable anymore, that is, it cannot be calculated based on purely observational data no matter how much data is collected. In the former case, the formula to calculate the effect changes and depending on the change between the correct formula for the true model and the incorrect formula based on the modified model more or less bias is induced on the effect estimate.

However, by now it remains unclear how large the probability is to reject a true structural causal model when data from it is observed. Thus, under ideal conditions – that is, when the correct causal relationships between the variables are reflected in the DAG representing the model – violations of the testable implications of the model can still produce contradictions to the hypothesized model structure. After observing such contradictions in some cases (which depend on the form of the contradiction) the DAG will be modified incorrectly and the resulting estimates of causal effects may suffer from introduced bias. In other cases, the causal model will be modified incorrectly and the causal effect of interest may become unidentified in the wrong modified model. In even other cases, the causal effect of interest may still be identified and although the causal model is modified incorrectly, the correct causal effect is still obtained correctly as its calculation does not depend on the falsely modified part of the causal model. All of these scenarios represent a kind of causal type I error, where the true model is rejected in favour of a false one. However, the severity of the three scenarios above differs substantially: For example, in the third case, the causal effect can still be obtained correctly, in the first case it becomes biased, and in the second case calculation of the causal effect of interest becomes entirely impossible. Thus, from a decision-theoretic perspective the loss associated with incorrectly rejecting a true causal model depends on the causal effect of interest and the structure of the model itself. All cases above are united by the fact that they describe some sort of *causal false-positive risk*, which corresponds to the probability of rejecting a true causal model based on its testable implications.

In this paper, two structural causal models which are widely used in medical research and epidemiology – confounders and colliders – are studied regarding their causal false-positive risk. Confounders and colliders play a central role in biomedical research, and more complex causal models often include these more elementary structures, so analyzing them first is helpful also to provide lower bounds on the causal false-positive risk of more elaborate and complex structural causal models. The plan of the paper is as follows: The next section reviews the basic definitions and properties of directed acyclic graphs (DAGs) and causal effects. Details about the building blocks of DAGs and identifiable structural coefficients are provided in the following section. There, the relevance of confounders and colliders for medical research is discussed. The section which follows analyzes possible violations of the testable implications of confounders and colliders, that is, which structural coefficients remain identified, and how the causal effect of interest changes depending on the set of violated testable implications. Then, a section discusses the goal and design of the simulation study which was carried out to (1) investigate the probability that the above causal models are rejected as false, although they reflect the true data-generating process, and (2) quantify the bias in estimating the structural coefficients and causal effects when the DAG is modified incorrectly. The following section discusses the results and the last section provides a conclusion.

## Methods

### Causal diagrams and directed acyclic graphs (DAGs)

This section provides a brief overview about causal diagrams and DAGs. To provide answers to causal questions from observational data, structural equation models (SEMs) are widely used in the data-intensive biomedical sciences. Such models allow to posit theoretical assumptions via structural equations, to derive their consequences and to put their statistical implications under test against the observed data. The resulting process is key to causal inference [1, 6] and a widely used representation of the model structure is given by *directed acyclic graphs (DAGs)*. DAGs can be obtained from a SEM by creating a *node* in the DAG for each variable in the SEM, and adding an *edge* between nodes depending on the relationships specified in the SEM equations. An edge between nodes *A* and *B* thus corresponds to a coefficient in the SEM, henceforth called a *structural coefficient*. Thus, DAGs allow to convert a SEM into a graphical representation, which has so-called *testable implications* [1]. Drawing causal inferences from a DAG then typically proceeds by checking the *identification* of the structural coefficients of interest (can the coefficient of interest be computed from observational data?), and reducing interventional probabilities by means of the do-calculus – see Pearl [1], Dawid [6] and VanderWeele [4, 7] – to observational probabilities. In sum, based on a DAG which represents a SEM, causal effects can be computed by well-established methods such as backdoor-adjustment [2], the front-door criterion [1], or counterfactual reasoning, based on *purely observational data*.

#### Directed acyclic graphs (DAGs)

We use the prototypical example of the effect of a treatment *X* on the outcome *Y* under the confounder *C*, the situation of which is shown in Fig. 1. The corresponding structural equation model

1 $$\begin{array}{*{20}l} &C=U_{C} \end{array} $$

2 $$\begin{array}{*{20}l} &X=\beta C + U_{X} \end{array} $$

3 $$\begin{array}{*{20}l} &Y=\alpha X + \gamma C + U_{Y} \end{array} $$

where *U*_*X*_,*U*_*C*_ and *U*_*Y*_ represent exogenous (error) variables which are not measured but which partially or entirely determine the value of the endogenous variables on the left-hand side above, and *α*,*β* and *γ* are the structural coefficients. In the DAG, such latent variables are distinguished from observed variables by being surrounded by a dashed box. The above model assumes that the choice of treatment is determined by *U*_*X*_ which is unobservable and could amount to personal preferences, severity of the disease, chance or a combination thereof, as well as the confounding variable *C*. The outcome *Y* is influenced by whether an individual chooses the treatment *X*, plus an additional disturbance *U*_*Y*_ as well as the confounding variable *C*. From Fig. 1 it is apparent that every variable in the model has a corresponding node in the graph. For each equation, an arrow is drawn from the independent variables on the right-hand side to the dependent variables on the left-hand side. These arrows reflect the direction of causation and after converting the variables and equations in a SEM into the nodes and edges of a DAG, the structural coefficients in the equations are appended to the corresponding arrows, in this case, the labels *α*,*β* and *γ*. A natural question is when a structural equation coefficient *α* yields information about the causal effect of treatment *X* on outcome *Y*. To estimate the causal effect *α* of *X* on *Y*, the coefficient must obey a unique solution in terms of the probability distribution of the observed variables *X*,*Y* and *C* (or equivalently, in terms of their covariance matrix). Identifying such a unique solution is called *identification of a causal effect*, and assuming linearity a linear regression model can provide a unique estimate for *α* in Fig. 1 based on least-squares estimation (after adjusting for the confounder *C*). In practice, however, it is often more difficult to identify a causal effect and sometimes it may even be unidentifiable based on the observable variables. The important implication is that no matter how many data one collects, it remains impossible to produce a point estimate for the value of an unidentifiable causal effect, that is, the corresponding structural coefficient(s).

Some definitions are required for spelling out sufficient criteria for a causal effect to be identifiable: An *edge* in a DAG is defined to be either an arrow or a bidirected arc. Whenever an arrow exists from *X* to *Y*, *X* is called a *parent* of *Y*, and when there exists a sequence of directed arrows from *X* to *Y*, then *Y* is called a *descendant* of *X*, and *X* an ancestor of *Y*. Nodes which are connected by bidirected arcs are called *siblings*. A *path* between *X* and *Y* is defined as a sequence of edges that connects the two nodes, and a path may be *directed* from *X* to *Y* (when every arrow points toward *Y*) or from *Y* to *X* (when every arrow points towards *X*). Important for the identification of causal effects are the notions of a *backdoor path*: A backdoor path from *X* to *Y* is any path which starts with an arrow pointing towards *X* and ends with an arrow pointing to *Y*. Also important is the notion of a collider: A *collider* is a node in which two arrowheads meet. Colliders block the flow of information, while backdoor paths are associated with confounding, for details see Pearl and MacKenzie [8] and Pearl [1]. Here, we consider only *acyclic* graphs, and an acyclic model without correlated error terms (the terms *U*_*X*_, *U*_*Y*_ in Fig. 1) is called *Markovian*. Whenever error terms are allowed to be correlated, the model is only *semi-Markovian*, and when the model becomes cyclic, the model is *non-Markovian*.

#### d-Separation

While in simple situations the identification of causal effects may be possible by visual inspection of the DAG, in more complex scenarios the property of *d*-separation is crucial. *d*-separation allows for more advanced criteria to identify causal effects, and also enables to test whether nodes corresponding to variables *Z* “block” a path from nodes in *X* to nodes in *Y*. *d*-separation is defined as follows, compare Pearl [1], p. 16:

##### **Definition 1**

(d-Separation) A path *p* is d-separated by a set of nodes *Z* if and only if

1. *p* contains a chain *i*→*m*→*j* or a fork *i*←*m*→*j* such that the middle node *m* is in *Z*, or
2. *p* contains a collider *i*→*m*←*j* such that the middle node *m* is not in *Z* and such that no descendant of *m* is in *Z*

A set *Z* d-separates *X* from *Y* if and only if *Z* d-separates every path from a node in *X* to a node in *Y*.

Thus, when *X* and *Y* given *Z* are *d*-separated, this stops the flow of information and it can be shown that this implies conditional independence of *X* and *Y* given *Z*, where the latter is denoted as *X*⊥ ⊥*Y*|*Z*, see Verma and Pearl [9] and Pearl [1], Chp.1.

##### **Theorem 1**

(Probabilistic Implications of *d*-Separation) If sets *X* and *Y* are *d*-separated by the set *Z* in a DAG *G*, then *X* is independent of *Y* conditional on *Z* in every distribution compatible with *G*. Conversely, if *X* and *Y* are not *d*-separated by *Z* in a DAG *G*, then *X* and *Y* are dependent conditional on *Z* in at least one distribution compatible with *G*.

Thus, *d*-separation is connected to the testable implications of a structural causal model which itself is represented by a DAG: Whenever *X* and *Y* are *d*-separated by the set *Z* in a DAG *G* it follows that *X*⊥ ⊥*Y*|*Z* in *G*. The latter is testable by inspection of the partial regression coefficient *β*_*YX*|*Z*_ of *X* on *Y* given *Z* in linear models, which should then equal zero, *β*_*YX*|*Z*_=0. This is seen from the relationship that *X*⊥ ⊥*Y*|*Z* implies *p*_*XY*|*Z*_=0 where *p*_*XY*|*Z*_ denotes the partial correlation coefficient between *X* and *Y* given *Z*, and the partial regression coefficient *β*_*YX*|*Z*_ is given as

4 $$\begin{array}{*{20}l} \beta_{YX|Z}=p_{YX|Z}\frac{\sigma_{{YZ}}}{\sigma_{{XZ}}} \end{array} $$

and reduces to zero under *p*_*XY*|*Z*_=0. Thus, *X*⊥ ⊥*Y*|*Z* implies *β*_*YX*|*Z*_=0 [1]. The second part of the above theorem is actually *“much stronger – the absence of d-separation implies dependence in almost all distributions compatible with G. The reason is that a precise tuning of parameters is required to generate independency along an unblocked path in the diagram, and such a tuning is unlikely to occur in practice.”*, for details see Pearl [1], p. 18 and Pearl [1], Corollary 5.2.2. This leads to the following more useful result in linear Markovian models (that is, linear models which admit no correlated error terms between variables), compare Verma and Pearl [9]:

##### **Theorem 2**

If sets *X* and *Y* are *d*-separated by *Z* in a DAG *G*, then *X* is independent of *Y* conditional on *Z* in every Markovian model structured according to *G*. Conversely, if *X* and *Y* are not *d*-separated by *Z* in a DAG, then *X* and *Y* are dependent conditional on *Z* in almost all Markovian models structured according to *G*.

Thus, when *X* and *Y* are not *d*-separated (that is, no set *Z*⊂*G* can be found which *d*-separates *Z* and *Y* in *G*), the dependency of *Y* and *X* holds in almost all distributions compatible with *G*. As a consequence, except for pathological cases where the precise tuning of parameters may generate independency along an unblocked path the testable implication is that the partial correlation coefficient *β*_*YX*|*Z*_ does not vanish, that is, *β*_*YX*|*Z*_≠0. Importantly, as stated by Pearl [1], Corollary 5.2.2, *“no other partial correlation would vanish”* except those whose variables are *d*-separated.

In the simulation study reported later, the simulation design ensures that such pathological cases do not occur, rendering the reverse part of the above theorem a helpful testable implication of a DAG.

Importantly, a Bayesian approach is taken in this paper to avoid the above pathologic cases. There are three primary reasons for using a Bayesian approach.

(i) First, Bayesian analysis has various advantages over traditional frequentist methods in biomedical research, for details see Kelter [10–12] and Wagenmakers et al. [13].
(ii) Second, null hypothesis significance tests aim at rejecting the null hypothesis *H*_0_:*β*=0 in favour of the alternative *H*_1_:*β*≠0. However, in a variety of testable implications such as *β*_*YX*|*Z*_=0, interest lies in *confirmation* of a null hypothesis. The Bayes factor allows to confirm such a null hypothesis as is discussed later.
(iii) Third, and most importantly, opting for a Bayesian approach to check the testable implications is important to ensure exclusion of the aforementioned pathological cases where *Y* is not conditionally independent of *X* given *Z* but the partial regression coefficients vanish, *β*_*YX*|*Z*_=0.

With regard to point (iii), while in the frequentist approach the true parameter is unknown and fixed, in the Bayesian paradigm the parameter is a random variable such as the observed data. Thus, under an absolutely continuous prior distribution *P*_*𝜗*_ with respect to the Lebesgue measure *λ* for the regression coefficients in a Bayesian linear regression model, the probability of the parameters (that is, the regression coefficients) taking the precise values which render an unblocked path independent (that is, *β*_*YX*|*Z*_=0, although *Y* is not conditionally independent of *X* given *Z*), is *zero* a priori. Thus, the assurance that such a tuning of parameters “is unlikely to occur in practice” is strengthened to the statement that such a tuning of parameters occurs with probability zero *P*_*𝜗*_-almost surely. This Bayesian perspective adds to the implication of the above Theorem that *“those (and only those) partial correlations identified by the d-separation test are guaranteed to vanish”* ([1], p. 142), the additive that “those (and only those) partial correlations not identified by the *d*-separation test are guaranteed *not* to vanish”. Opting for a Bayesian statistical analysis thus allows to make the “almost all Markovian models” part in Theorem 2 explicit through the prior distribution *P*_*𝜗*_.

It is worth noting from a computational point of view that next to manual inspection whether *X* and *Z* are *d*-separated by a set *Z* based on Definition 1, there are also algorithms which allow for automating this process, see Lauritzen et al. [14] and Dawid [6].

#### Causal effects

After a structural equation model has been converted to a DAG, a precise definition of the causal effect of *X* on *Y* is required. Therefore, let $\mathcal {P}:=\{p_{1},...,p_{n}\}$ be the set of directed paths from *X* to *Y* and *p*_*i*_ denote the product of structural coefficients along path *p*_*i*_. The *total effect* or *average causal effect (ACE)* according to Bollen [15] is given as

5 $$\begin{array}{*{20}l} \text{ACE}(X,Y):=\sum_{i=1}^{n} p_{i} \end{array} $$

For example, in Fig. 1, *ACE*(*X*,*Y*)=*α*. This “path-tracing” definition goes back to Wright [16] and in linear systems coincides with the expected value of a variable *Y*, after *X* is assigned the value *x* by intervention, denoted $\mathbb {E}[Y|do(X=x)]$, for details see Chen and Pearl [17], p. 4. The difference between conditioning on *X*=*x* and setting *X*=*x* by intervention, denoted as *do*(*X*=*x*) is important for causal inference and the associated *do-calculus* is outlined in detail in Pearl [1], Chapter 1-5, see also Dawid [6] and Berzuini et al. [18]. Thus, ACE can be expressed equivalently as

6 $$\begin{array}{*{20}l} \text{ACE}(X,Y)=\frac{\partial}{\partial x}\mathbb{E}[Y|do(X=x)] \end{array} $$

In many cases however, one is interested in the *direct effect* of *X* on *Y*. The direct effect of *X* on *Y* is the sensitivity of *Y* to changes in *X* while all other factors in the analysis are held fixed, see Pearl [1], Chp. 4.5. The process of holding all other factors fixed can be conceptualized in a DAG as a “graph surgery” in which all arrows from parents of *X* which run into *X* are severed, leaving only direct links from *X* to *Y* (compare Figs. 1 and 2, where randomization achieves the same, which is why an RCT can answer causal questions).

Direct effects can be calculated from do-free expressions and evaluated via purely observational data under the conditions given in Pearl ([1], Definition 4.5.1, Theorem 4.5.3), see also Pearl et al. [2], p. 77. The single-door criterion provides means to calculate a direct effect:

##### **Theorem 3**

(Single-door Criterion) Let *G* be any acyclic causal graph in which *α* is the coefficient associated with arrow *X*→*Y*, and let *G*_*α*_ denote the graph which results when *X*→*Y* is deleted from *G*. The coefficient *α* is identifiable if there exists a set of variables *Z* such that

(i) *Z* contains no descendant of *Y* and
(ii) *Zd*-separates *X* from *Y* in *G*_*α*_

If *Z* satisfies these two conditions, then *α*is equal to the regression coefficient *β*_*YX*|*Z*_. Conversely, if *Z* does not satisfy these conditions, *β*_*YX*|*Z*_ is not a consistent estimate of *α*.

A proof of the single-door criterion is given in Pearl [19] and Spirtes et al. [20], see also Pearl [1], Chp. 5. The backdoor criterion allows to identify the average causal effect based on *d*-separation between nodes in a DAG:

##### **Theorem 4**

(Back-door Criterion) For any two variables *X* and *Y* in a causal diagram *G*, the total effect of *X* on *Y* is identifiable if there exists a set of variables *Z* such that

(i) no member of *Z* is a descendant of *X*
(ii) *Zd*-separates *X* from *Y* in the subgraph $G_{\underline {X}}$ formed by deleting from *G* all arrows starting in *X*.

If the two conditions are satisfied, the total effect of *X* on *Y* is given by the regression coefficient *β*_*YX*|*Z*_.

Details are provided in Pearl [1], Chapter 3.3.1.

## Confounders and colliders

The previous section outlined the basic theoretical results which are required for a detailed analysis of confounder and collider models. As is inevitably the case, every inferential situation can result in its own specific DAG which makes it impossible to provide results for the causal false-positive risk independent of the underlying structural model. However, in the majority of research there are key components which could be called “causal bricks” that are frequently observed and together can build larger and more complex graphs. In the context of biomedical research and epidemiology, two of these typical causal bricks are confounders and colliders (next to mediators and confounded mediators), see VanderWeele [4, 7], Dawid [6], Pearl et al. [2] and Hernán and Robins [21]. In this section, we assume a linear Markovian model (that is, acyclic and uncorrelated error terms) and consider the prototypical situation of the effect of a treatment *X* on the outcome *Y*.

### Confounders

**Fig. 3:**
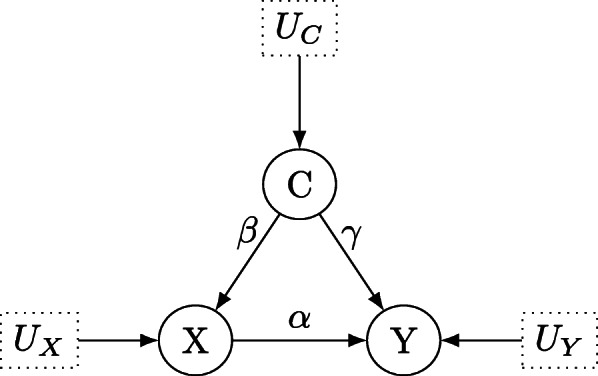
Directed acyclic graph for the structural causal model which describes the effect of treatment *X* on outcome *Y* under the confounding variable *C*. *U*_*X*_, *U*_*Y*_ and *U*_*C*_ denote unobservable latent variables, which determine the values of *X*, *Y* and *C*

The first causal brick is the situation where the effect of the treatment *X* on the outcome *Y* is confounded by a confounding variable *C*, displayed in Fig. 3. The structural coefficients are shown as *α*,*β* and *γ* and we first turn to the testable implications of this model. Clearly, no variable is *d*-separated conditional on any other set of variables in Fig. 3: For example, *Y*⊥ ⊥*X*|*C* does not hold as the path *X*→*Y* remains unblocked after conditioning on *C*. Thus, although the path *X*←*C*→*Y* is blocked by *C*, the nodes *X* and *Y* are not *d*-separated by *C*. Analogue arguments show that *Y* ⊥̸ ⊥*C*|*X* and *X* ⊥̸ ⊥*C*|*Y*. Note that *Y* is a collider in the path *C*→*Y*←*X*, and thus *Y* blocks this path. *X* ⊥̸ ⊥*C* holds even without conditioning on *Y*: the path *C*→*X* stays open, regardless on whether one conditions on the collider *Y* additionally, opening the path *C*→*Y*←*X* or not. The symmetry property of conditional independence – that is, *A* ⊥̸ ⊥*B*|*C*⇔*B* ⊥̸ ⊥*A*|*C*, compare Dawid [6] – shows that we cannot *d*-separate any nodes in Fig. 3 (conditioning on the empty set *∅* clearly also does not *d*-separate any nodes in Fig. 1). As outlined in the previous section, no partial correlation coefficients are expected to vanish, and due to the arguments below Theorem 2 we obtain the testable implications that

7 $$\begin{array}{*{20}l} p_{YX|C}\neq 0, \hspace{0.5cm} p_{YC|X}\neq 0, \hspace{0.5cm} p_{XC|Y}\neq 0 \end{array} $$

In the simulation study, we will use these constraints to estimate the resulting causal false-positive rate and the bias in estimating the causal effect of treatment *X* on outcome *Y*. As noted above, *X* ⊥̸ ⊥*C* which we use to employ the constraint *p*_*XC*_≠0 instead of *p*_*XC*|*Y*_≠0. This avoids biased estimates of the regression coefficient *β*_*XC*|*Y*_ by opening the collider *Y* in *C*→*Y*←*X* later. Now, the testable implications are given as

8 $$\begin{array}{*{20}l} \beta_{YX|C}\neq 0, \hspace{0.5cm} \beta_{YC|X}\neq 0, \hspace{0.5cm} \beta_{{XC}}\neq 0 \end{array} $$

Turning to the identification of causal effects under confounding as shown in Fig. 3, the backdoor-criterion as given in Theorem 4 shows that *C* is a backdoor-admissible set and the total effect ACE(X,Y)=*α* (there is only a single directed path from *X* to *Y* in the DAG, and no other path from *X* to *Y*, and thus it also follows that IE(X,Y)=0) of treatment *X* on outcome *Y* can be estimated by the (unbiased) estimand *β*_*YX*|*C*_,

9 $$\begin{array}{*{20}l} \text{ACE}(X,Y)=\beta_{YX|C} \end{array} $$

As there is only one directed arrow from *X* to *Y* in Fig. 3, it is immediate that there is no indirect effect of *X* on *Y*, and thus the direct effect is equal to the total effect, as in linear systems, the equality

10 $$\begin{array}{*{20}l} \text{ACE}(X,Y)=\text{DE(X,Y)}+\text{IE(X,Y)} \end{array} $$

holds, compare Pearl [1], Section 4.5.5 and Pearl et al. [2], p. 83-87. Therefore,

11 $$\begin{array}{*{20}l} &\text{DE}(X,Y)=\beta_{YX|C} \end{array} $$

12 $$\begin{array}{*{20}l} &\text{IE}(X,Y)=0  \end{array} $$

### Colliders

**Fig. 4:**
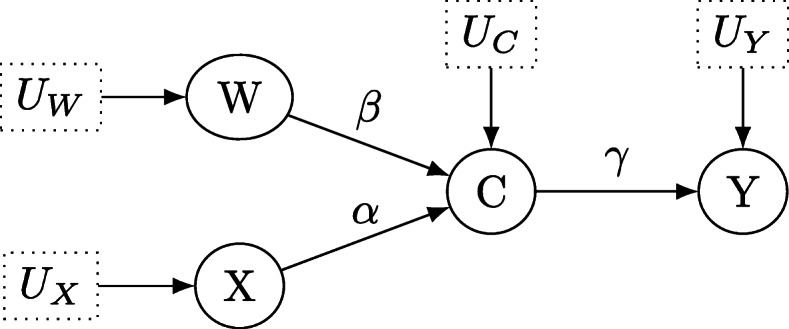
Directed acyclic graph for the structural causal model which describes the effect of treatment *X* on outcome *Y* under the collider variable *C*. *U*_*X*_, *U*_*Y*_, *U*_*W*_ and *U*_*C*_ denote unobservable latent variables

The second causal brick considered is the frequently observed setting of a collider as shown in Fig. 4. The treatment *X* has a direct causal effect on the collider *C*, which is also influenced by another variable *W*. For example, the treatment *X* could now be a drug for lowering blood pressure that affects physiological properties *C*. However, *W* could represent whether an individual exercises regularly which also affects *C*, and *C* itself influences the outcome *Y* which could be cardiac failure. Importantly, neither *W* nor *X* have a direct cause on *Y*, but the causal effects are transmitted through the collider *C* in *W*→*C*←*X*. Note that conditioning on *C* correlates the previously uncorrelated variables *X* and *C*: Individuals having a specific value of *C*, e.g. risk-reducing physiological properties, will either pick an appropriate dose of the treatment, and thus require no additional exercising, or will exercise a lot and thus require no (or little) additional treatment *X*. Thus, given *C*=*c*, *W* and *X* become (negatively) correlated.

Inspecting the testable implications of Fig. 4, it is apparent that *Y* ⊥ ⊥*X*|*C* and *Y* ⊥ ⊥*W*|*C*, as *C* blocks every path from *X* to *Y* or *W* to *Y* (compare Definition 1). Also, *W* ⊥ ⊥*X* as the empty set *∅* blocks every path between *W* and *X* in Fig. 4: *X* and *W* are separated by the collider *C* in *X*→*C*←*X*. However, *W* ⊥̸ ⊥*X*|*C* as conditioning on the collider *C* opens the only path *W*→*C*←*X* between *W* and *X*. This yields the testable implications

13 $$\begin{array}{*{20}l} &p_{YX|C}= 0, \hspace{0.5cm} p_{YW|C}= 0, \end{array} $$

14 $$\begin{array}{*{20}l} &p_{{WX}}=0, \hspace{0.5cm} p_{WX|C}\neq 0 \end{array} $$

which are equivalent to

15 $$\begin{array}{*{20}l} &\beta_{YX|C}= 0, \hspace{0.5cm} \beta_{YW|C}= 0, \\ &\beta_{{WX}}=0, \hspace{0.5cm} \beta_{WX|C}\neq 0 \end{array} $$

Furthermore, as *C* is not *d*-separated from *W* by {*X*,*Y*} we can conclude that *C* ⊥̸ ⊥*W*|{*X*,*Y*} and thus *p*_*CW*|{*X*,*Y*}_≠0, *P*_*𝜗*_-almost surely in almost all Markovian linear models (compare Theorem 2), which in turn implies *β*_*CW*|{*X*,*Y*}_≠0. Likewise, because *C* is not *d*-separated from *X* given {*W*,*Y*}, it follows that *β*_*CX*|{*W*,*Y*}_≠0. Finally, *Y* ⊥̸ ⊥*C*|{*W*,*X*}, and thus also *β*_*YC*|{*W*,*X*}_≠0, so that Fig. 4 yields the additional testable implications

16 $$\begin{array}{*{20}l} \beta_{CW|\{X,Y\}}\neq 0, \beta_{CX|\{W,Y\}}\neq 0, \beta_{YC|\{W,X\}}\neq 0 \end{array} $$

Concerning the total effect of *X* on *Y*, we obtain

17 $$\begin{array}{*{20}l} \text{ACE}(X,Y)=\sum_{i=1}^{1} p_{i}=\gamma\alpha \end{array} $$

as *p*_1_:=*X*→*C*→*Y*. Clearly, there is no direct arrow from *X* to *Y* in Figure 4, so

18 $$\begin{array}{*{20}l} \text{DE}(X,Y)=0 \end{array} $$

and from Eq. (10) it follows that

19 $$\begin{array}{*{20}l} \text{IE}(X,Y)&=\text{ACE}(X,Y)-\text{DE}(X,Y)  \\ &=\text{ACE}(X,Y)=\alpha\gamma \end{array} $$

From Theorem 3 it follows that *∅* is a single-door admissible set for estimating *α* by *β*_*CX*_. Likewise, *γ* is estimable as *β*_*YC*_ due to the same reason. It thus follows that the estimand for ACE(X,Y)=IE(X,Y) is given as

20 $$\begin{array}{*{20}l} \text{ACE(X,Y)}=\text{IE(X,Y)}=\beta_{{CX}}\cdot \beta_{{YC}} \end{array} $$

### Testable implications and graph modifications

In this section, we pose the question what happens when one or multiple of the testable implications are falsified, although the true data-generating model corresponds to one of the two causal models described in the above section.

#### Testable implications of confounders

Return to Fig. 3: Suppose that we observe data *X*,*Y* and *C* and set up two Bayesian linear regression models by regressing *Y* on *X* and *C*, and by regressing *X* on *C*. Then, *β*_*YX*|*C*_ and *β*_*YC*|*X*_ are obtained as the regression coefficients for *X* and *C* in the first model, while *β*_*XC*_ is obtained as the corresponding regression coefficient in the second regression model. The testable implications in Eq. (8) can now be checked as follows: Suppose the hypothesis tests for *H*_0_:*β*=0 against *H*_1_:*β*≠0 for all partial regression coefficients *β* given in Eq. (8) show evidence for all three coefficients being equal to zero. For example, these tests could be conducted by checking whether the Bayes factor *BF*_01_ of the hypothesis *H*_0_:*β*=0 against the alternative *H*_1_:*β*≠0 passes a predefined threshold, compare Kelter [22]. The resulting modification of the DAG would correspond to deleting all arrows between *X*,*Y* and *C*, and thus it would follow that ACE(X,Y)=DE(X,Y)=IE(X,Y)=0.

Suppose on the other hand that only two hypothesis tests confirm that a regression coefficient is zero.

- When *β*_*YX*|*C*_=0 and *β*_*YC*|*X*_=0, we also have ACE(X,Y)=DE(X,Y)=IE(X,Y)=0 because we would erase both arrows from *C*→*Y* and from *X*→*Y*.
- When *β*_*YX*|*C*_=0 and *β*_*XC*_=0, we would also have ACE(X,Y)=DE(X,Y)=IE(X,Y)=0, because we would erase the arrow *C*→*X* and the arrow *X*→*Y*, thus deleting all paths between *X* and *Y*.
- When *β*_*YC*|*X*_=0 and *β*_*XC*_=0, we would not identify *C* as a confounder anymore via the backdoor-criterion, and compute ACE(X,Y) without conditioning on *C*. Thus, we would arrive at ACE(X,Y)=*β*_*YX*_ which is obtained from the newly built regression model which regresses *Y* on *X* only, and obtain DE(X,Y)=*β*_*YX*_=ACE(X,Y). Thus, both the average causal and direct effect are biased in this case.

Suppose now that only a single of the three hypothesis tests confirms that a regression coefficient is zero:

- If *β*_*YX*|*C*_=0, we would erase the arrow from *X*→*Y* and obtain ACE(X,Y)=DE(X,Y)=IE(X,Y)=0.
- If *β*_*YC*|*X*_=0, we would erase the arrow from *C*→*Y* and obtain ACE(X,Y)=DE(X,Y)=*β*_*YX*_ from the newly built regression model of *Y* only on *X* (*Y* is conditionally independent of *C* given *X* in the modified graph)
- If *β*_*XC*_=0, we would erase the arrow from *C*→*X* and obtain ACE(X,Y)=DE(X,Y)=*β*_*YX*_ from the newly built regression model of *Y* only on *X*, because *Z*:=*∅* satisfies the backdoor-criterion as *X* and *Y* are *d*-separated by *∅* in the subgraph *G*_*α*_ when the arrow *C*→*X* is removed from Fig. 3.

Thus, while all of the above cases contribute to the causal false-positive risk under the situation depicted in Fig. 3, the bias which is implied for the average causal, direct and indirect effects ACE(X,Y), DE(X,Y) and IE(X,Y) differs depending on the actual outcome of the performed hypothesis tests to check the testable implications of the underlying model. In sum, the above reasoning leads to the following result:

##### **Lemma 1**

Assume the true structural causal model is given by the directed acyclic graph *G* in Fig. 3 with testable implications given in Eq. (8). The induced bias on ACE(X,Y), DE(X,Y), IE(X,Y) for each possible violation of one or multiple testable implications (each case of which contributes to the causal-false positive risk) are given in Table 1 in the Supplementary file.

##### *Proof*

See Supplementary file. □

#### Testable implications of colliders

Now, consider the testable implications of colliders as depicted in Fig. 4. The implications in Eq. (15) and (16) are more complex than for the case of confounding shown in Fig. 3, but with the exception of a single testable implication, checking the testable implications and deriving the consequences of violations of any of them is straightforward.

We consider the single testable implication which requires more attention first, that is, *β*_*WX*|*C*_≠0. We discuss this implication in the context of the testable implication *β*_*WX*_=0, and show that it can be removed, leaving only six testable implications

21 $$\begin{array}{*{20}l} &\beta_{YX|C}= 0, \hspace{0.5cm} \beta_{YW|C}= 0, \hspace{0.5cm} \beta_{CW|\{X,Y\}}\neq 0 \\ &\beta_{WX}=0, \hspace{0.25cm} \beta_{CX|\{W,Y\}}\neq 0, \hspace{0.30cm}\beta_{YC|\{W,X\}}\neq 0 \end{array} $$

remaining. Therefore, consider the case when *β*_*WX*_=0: Then, no directed path from *W* to *X* or from *X* to *W* exists (possibly including other nodes in the graph). Assume that the testable implication *β*_*WX*|*C*_≠0 now is violated, which implies *β*_*WX*|*C*_=0, then *C* cannot be a collider on the path *W*→*C*←*X* anymore. The following options exist for this scenario:

- Option 1: *W*→*C*→*X*, then we have *β*_*WX*|*C*_=0, but reversing the direction of the arrow *X*→*C* to *C*→*X* means that the treatment *X* has no causal effect on *C* anymore (e.g. parameters in a blood sample). However, this contradicts the causal assumption how the treatment *X* works, and as data are purely observational the treatment *X* (which often corresponds to taking some drug or applying some kind of intervention) has succeeded in a long administration process via clinical studies. Next to contradicting these extra-mathematical arguments, assuming the path *W*→*C*→*X* also contradicts the assumption *β*_*WX*_=0, as then *W* and *X* are clearly correlated (through *C*).
- Option 2: *X*→*C*→*W*, which also is rendered unrealistic as *W* could stand for gender, which has an effect on the blood sample parameters *C*. Thus, as *C* cannot change the gender *W*, this assumption is also rendered unrealistic. Even when *W* corresponds to some other variable, say, physical activity, *X*→*C*→*W* contradicts the assumption *β*_*WX*_=0, too.
- Option 3: Erase both arrows *X*→*C* and *W*→*C*, then *β*_*WX*|*C*_=0, but neither the treatment *X* nor the other variable *W* have any causal influence on *C* then anymore, which is unrealistic due to the same arguments brought forward for Option 1.
- Option 4: Erase the arrow *X*→*C*. This implies *β*_*WX*|*C*_=0 but is unrealistic, too, due to the same arguments brought forward for Option 1.
- Option 5: Erase the arrow *W*→*C*: Then, *β*_*WX*|*C*_=0 and *β*_*WX*_=0. In this last case, the treatment still has a causal effect on *C* but the influence of *W* is questioned.

In sum, except for Option 5 all other options are either contradicting *β*_*WX*_=0 or are rendered implausible due to extra-mathematical arguments. Thus, whenever *β*_*WX*_=0 holds and *β*_*WX*|*C*_≠0 is violated, the consequence is to modify the original DAG by erasing the arrow from *W* to *C* with label *β*. Thus, this two-stage test via *β*_*WX*_ and *β*_*WX*|*C*_ is an implicit test for the existence of the arrow *W*→*C* in the DAG, and such a test is already existent in the set of testable implications through the testable implication *β*_*CW*|{*X*,*Y*}_. Thus, the two-stage test *β*_*WX*_ and *β*_*WX*|*C*_ is not necessary in the above case and can be replaced by the test of *β*_*CW*|{*X*,*Y*}_ when *β*_*WX*_=0 holds and *β*_*WX*|*C*_≠0 is violated.

What about the other cases? When *β*_*WX*_=0 holds and *β*_*WX*|*C*_≠0 is not violated, neither constraint is violated and the DAG stays the same.

When *β*_*WX*_=0 is violated, there must be a direct path between *W* and *X* in the DAG (excluding the case where *X*←*C*→*W* because then the treatment has no effect on *C* anymore). The cases *W*→*C*→*X* and *X*→*W*→*C* are thus excluded because they are again unrealistic as they imply that *X* has no effect on *C* anymore or that *C* can affect *W* (which could stand for gender, but even when *W* is physical activity, a different set of blood parameters will not *cause* higher or lower physical activity; it could possibly allow for a better health situation, which would add a new node in the DAG, that then influences *W*). The remaining two cases are to add an arrow *X*→*W* or *W*→*X* to the DAG. These are narrowed down to *W*→*X*, because as mentioned above, *X* will neither change the gender *W* of an individual, nor have a direct causal effect on the physical activity (any such effect would be mediated by health status, motivation, quality of life, et cetera of a patient which are affected by the treatment).

**Fig. 5:**
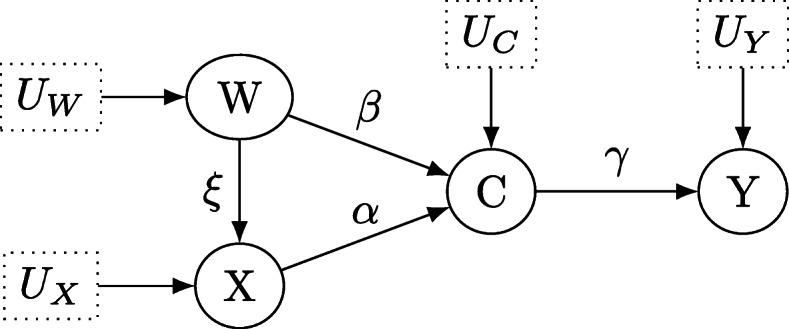
Directed acyclic graph for the structural causal model which describes the effect of treatment *X* on outcome *Y* under the collider variable *C*. *U*_*X*_, *U*_*Y*_, *U*_*W*_ and *U*_*C*_ denote unobservable latent variables. The arrow connecting *W* and *X* is assumed to be directed from *W* to *X* and not from *X* to *W*, either due to subject-domain knowledge or temporal information

In sum, the only option is to add the arrow *W*→*X* to the DAG (resulting in the modified DAG shown in Fig. 5) whenever *β*_*WX*_=0 is violated, and importantly, as then *β*_*WX*_≠0, the testable implication *β*_*WX*|*C*_≠0 is then *always true*: Conditioning on *C* will not *d*-separate *W* from *X* because of the direct path *W*→*X*, which cannot be closed.

In total, in every case the test of *β*_*WX*|*C*_≠0 is superfluous. No information is gained whenever *β*_*WX*_=0 is violated. Whenever *β*_*WX*_=0 and *β*_*WX*|*C*_≠0 hold and neither testable implication is violated it implies no change to the DAG or it can be replaced by the check of *β*_*CW*|{*X*,*Y*}_. Thus, the testable implication *β*_*WX*|*C*_≠0 can be removed and the remaining testable implications are given by Eq. (21).

##### **Assumption 1**

Let *G* be the directed acyclic graph in Fig. 4 corresponding to the collider situation, and suppose that the testable implication *β*_*WX*_=0 is violated. Then, a directed arrow *W*→*X* is added to the DAG which is justified either by subject-domain knowledge or temporal information.

Each of these remaining six testable implications has an unambiguous consequence when being violated. For example, when *β*_*YW*|*C*_=0 is violated, an arrow from *X* to *Y* is added to the DAG, because (again borroughing the example where *W* stands for gender) the variable *W* may have a direct causal influence on the outcome *Y*, but the opposite can often be questioned. In cases, where the directionality of the arrow is questionable (e.g. when *W* is physical activity), it is assumed that temporal information is available which allows to specify the direction *W*→*Y* (that is, the observational data allow to judge that patients have not become physically more or less active through the outcome; this is often straightforward, because such an effect would again be transmitted through a change in motivation, pain reduction, or other variables which would correspond to new nodes in the DAG, so that no direct arrow would be drawn from *Y* to *W*).

The above analysis also showed that when *β*_*WX*_=0 is violated, an arrow from *W* to *X* and not from *X* to *W* is inserted into the DAG, and when for example *β*_*CX*|{*W*,*Y*}_≠0 is violated, the arrow *X*→*C* would be deleted from the DAG.

**Fig. 6:**
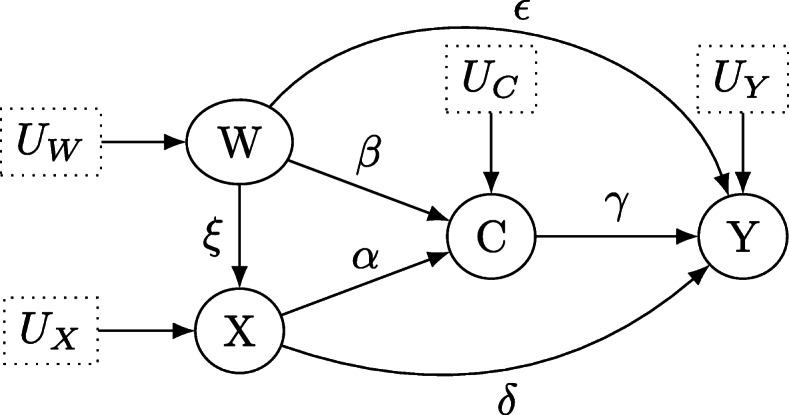
Directed acyclic graph for the structural causal model which describes the effect of treatment *X* on outcome *Y* under the collider variable *C*. *U*_*X*_, *U*_*Y*_, *U*_*W*_ and *U*_*C*_ denote unobservable latent variables. The presence of each of the arrows depends on which of the testable implications for the collider scenario are violate

In total, the six testable implications can thus be violated in the following structured ways: Either, a single implication is violated, or exactly two implications are violated, or exactly three are violated, or exactly four, or exactly five, or exactly six. The number of these violations is given as $\sum _{k=1}^{6} {6\choose k} =6+15+20+15+6+1=63$, and in a large number of cases the direct, indirect and average causal effects will reduce to zero immediately. The Supplementary file outlines each of these cases and how the estimands for ACE(X,Y), DE(X,Y) and IE(X,Y) change for each case. The results allow to test for any possible violations of the testable implications and show which of the arrows in Fig. 6 is existent in the modified DAG in each case. Whether one or multiple arrows are deleted in Fig. 6 depends on which of the six testable implications in Eq. (21) are violated. The above line of thought thus leads to the following result:

##### **Lemma 2**

Assume the true structural causal model is given by the directed acyclic graph *G* in Fig. 4 with testable implications given in Eq. (21) and suppose Assumption 1 holds. The induced bias on ACE(X,Y), DE(X,Y), IE(X,Y) for each possible violation of one or multiple testable implications (each case of which contributes to the causal-false positive risk) is then given in Tables 2 and 3 in the Supplementary file.

##### *Proof*

See Supplementary file. □

### Simulation study

Now, based on the previous section it is clear that the simulation study mimicks a smart investigator who adaptively responds to violations of the testable implications of his causal model. The first goal of the simulation study is to investigate the causal false-positive risk, that is, to provide answers to question (*i*) below.

(i) How large is the probability to reject a true structural causal model represented as a DAG based on its testable implications?
(ii) How large is the resulting bias for estimates of average causal effects, direct and indirect causal effects based on falsely rejecting a true structural causal model which is represented as a DAG?

Question (ii) goes hand in hand with question (i), because bias can only occur when the true estimands for ACE(X,Y), DE(X,Y) and IE(X,Y) change. The latter happens if and only if the true structural causal model is rejected, which in turn can only be the case whenever one or multiple of the testable implications of the DAG are violated.

Naturally, the answers to the above questions will depend on the statistical methods to check the testable implications, the magnitude of the true structural coefficients, and the amount of data available to the researcher.

#### Statistical analysis of the testable implications

Statistical analysis of the testable implications proceeds via standard linear regression analysis. As we operate in linear Markovian models (uncorrelated error terms, no cycles), the standard regression coefficients *β*_*AB*_ of regressing *A* on the independent variable *B*, or the partial regression coefficients *β*_*AB*|*C*_ which equal the regression coefficient of the variable *B* in the multiple linear regression model of *Y* on both *A* and *B* provide the estimands which are subsequently used to check the testable implications. As mentioned already above, a Bayesian approach is taken in this paper, compare the section on d-separation, which analyzes the testable implications by means of standard Bayesian linear regression models (for an overview see Van Erp et al. [23], Robert [24] or Kruschke [25]). The Bayes factor *BF*_01_ in favour of the null hypothesis *H*_0_:*β*=0 against *H*_1_:*β*≠0 is used to test the conditional independencies and dependencies which are given as the testable implications of each of the models. The Bayes factor measures the relative change in beliefs towards either of the hypotheses under consideration, and under equal prior probabilities for *H*_0_:*β*=0 and *H*_1_:*β*≠0 equals the posterior probabilities for *H*_0_ and *H*_1_. Thus, the threshold *BF*_01_>1 provides a natural criterion for a violation of the testable implication *β*≠0, and *BF*_01_<1 implies a violation of the testable implication *β*=0. In the former case, the posterior probability (under equal prior weights for *H*_0_ and *H*_1_) indicates that *H*_0_:*β*=0 is more probable given the data, and in the latter case, the posterior probability shows that *H*_1_:*β*≠0 is more probable than *H*_0_:*β*=0 after observing the data. Details about the Bayes factor are provided in Robert [24], Wagenmakers et al. [13] and Kelter [11, 22] as well as Berger [26] and Schervish [27].

It is important to note that there are various alternative statistical evidence measures to the Bayes factor. An overview is provided by Kelter [28], and Makowski et al. [29], Linde et al. [30] and Kelter [10, 31, 32] have shown that different Bayesian approaches to hypothesis testing can yield varying conclusions for identical data. As the Bayes factor is one of the most widely established approaches to hypothesis testing in medical research [11, 13, 33], in this paper the focus is on using a single evidence measure which is the Bayes factor. This allows to compare the results and also enables to use relationships between Bayes factors and p-values which have been established in the statistical literature [34, 35]. However, future research should also investigate the dependency of the results obtained here on the evidence measure which is employed for testing the hypotheses corresponding to the testable implications.

Next to the choice how to test a hypothesis the role of the prior is crucial in a Bayesian analysis. Here, weakly-informative normal priors are employed on the regression coefficients, following the recommendation of Goodrich [36], see also the section on the simulation design. As stressed by Gelman et al. [37], using such priors in hierarchical models provides a natural Bayesian type I error control.

#### Local and global testing

A short note should be made on the strategy of local over global testing. As stressed by Pearl [1], the approach to formalize all testable implications in terms of the covariance matrix of the resulting model and perform a global test on the covariance matrix as a whole turns out problematic in a variety of cases: Researchers usually want to modify their causal model represented as a DAG when one or multiple of the testable implications are violated. Hence, a global test can only invalidate the set of all testable implications as a whole but does not provide hints where the underlying structural causal model may be misspecified, and refinement is needed.

Additionally, some structural coefficients may not be identified which implies that in some cases a global test of the testable implications cannot be performed at all because of missing entries in the covariance matrix. In contrast, local tests may still be possible through the use of instrumental variables (see Chen and Pearl [17], p. 10 and Pearl [1]):

“Global tests represent summaries of the overall model-data fit and, as a result, violation of specific testable implications may be masked (Tomarken and Waller, 2003). In contrast, if the testable implications are enumerated and tested individually, the model can be tested even when unidentified, the power of each test is greater than that of a global test (Bollen and Pearl, 2013; McDonald, 2002), and in the case of failure, the researcher knows exactly which constraint was violated.” Chen and Pearl [17], p. 16

As a consequence, the approach taken in this paper focusses on local over global testing of the model constraints and testable implications. Furthermore, this is precisely what allows to simulate a researcher in the simulation study who adaptively modifies his structural causal model by deleting or adding arrows in his DAG, depending on the set of violated testable implications based on the observed data.

#### Magnitude of the structural coefficients

Consider the situation in Fig. 3 which depicts the confounder scenario. The resulting causal false-positive risk, that is, the probability of violation of one or multiple of the testable implications in Eq. (8) depends on the magnitude of the structural coefficients *α*,*β* and *γ*. First, when the relationships between *X*,*Y* and *C* are too strong or self-evident, it follows that none of the testable implications will be violated, even for small sample sizes. Thus, the magnitude of *α*,*β* and *γ* must not be too large. Suppose *α*:=0.25. Then, we can separate between different magnitudes of the confounder *C*. Consider a balanced confounder, that is *β*=*γ*. The influence on *X* and *Y* could be as strong as the influence of *X* on *Y*, yielding *β*=*γ*=0.25. This situation, henceforth called balanced leveled confounder, builds the starting point in the simulations. Next to this setting, the confounder could exert considerably stronger or weaker influence on *X* and *Y*: For example, setting *β*=*γ*=0.125 amounts to a confounder which exerts half of the influence on *X* and *Y* as *X* exerts on *Y*. On the contrary, *β*=*γ*=0.5 or *β*=*γ*=1 correspond to confounders which exert twice or four times as much influence on *X* and *Y* as *X* exerts on *Y* itself. We study all of these four settings, and additionally consider also unbalanced confounders.

Natural choices are given by *β*=0.25 and *γ*=0.75 as well as *β*=0.75 and *γ*=0.25. More extreme cases of unbalanced confounders amount to *β*=0.1 and *γ*=0.9 as well as *β*=0.9 and *γ*=0.1. These two settings are investigated as well, and in each setting the testable implications in Eq. (8) serve as the basis for conducting local Bayesian hypothesis tests via the Bayes factor, and consecutively adapting the true DAG in Fig. 3 according to the rules outlined in earlier. In case of any violations of the testable implications, the resulting estimands for *ACE*(*X*,*Y*), *DE*(*X*,*Y*) and *IE*(*X*,*Y*) thus change as specified in the section on the testable implications of confounders. Table 1 provides an overview about the simulation settings for confounders.

**Table 1:** Simulation settings for confounders (Fig. 3)

Setting	*α*	*β*	*γ*
Balanced			
Weak Confounder	0.25	0.125	0.125
Balanced Confounder	0.25	0.25	0.25
Strong Confounder	0.25	0.5	0.5
Very strong Confounder	0.25	1.0	1.0
Weak Confounder	0.5	0.125	0.125
Balanced Confounder	0.5	0.25	0.25
Strong Confounder	0.5	0.5	0.5
Very strong Confounder	0.5	1.0	1.0
Unbalanced			
Unbalanced Confounder	0.25	0.25	0.75
Unbalanced Confounder	0.25	0.75	0.25
Unbalanced Confounder	0.25	0.1	0.9
Unbalanced Confounder	0.25	0.9	0.1
Unbalanced Confounder	0.5	0.25	0.75
Unbalanced Confounder	0.5	0.75	0.25
Unbalanced Confounder	0.5	0.1	0.9
Unbalanced Confounder	0.5	0.9	0.1

Consider the situation in Fig. 4. First, balanced colliders are considered, that is *α*=*β*=*γ*=0.25. Then, *α*=*β*=0.5 and *α*=*β*=1 are considered for *γ*=0.25. Also, the above settings are repeated for *γ*=0.5 and *γ*=1. In addition, unbalanced colliders are considered: *α*=1/3, *β*=2/3 for *γ*=0.25 and *α*=2/3, *β*=1/3 likewise. Also, *α*=0.1, *β*=0.9 and *α*=0.9, *β*=0.1 are considered, too. These unbalanced settings are then again repeated for *γ*=0.5 and *γ*=1. Table 2 provides an overview about the simulation settings for colliders.

**Table 2:** Simulation settings for colliders (Fig. 4)

Setting	*α*	*β*	*γ*
Balanced	0.25	0.25	0.25
Balanced	0.5	0.5	0.25
Balanced	1.0	1.0	0.25
Balanced	0.25	0.25	0.5
Balanced	0.5	0.5	0.5
Balanced	1.0	1.0	0.5
Balanced	0.25	0.25	1.0
Balanced	0.5	0.5	1.0
Balanced	1.0	1.0	1.0
Unbalanced	1/3	2/3	0.25
Unbalanced	2/3	1/3	0.25
Unbalanced	1/3	2/3	0.5
Unbalanced	2/3	1/3	0.5
Unbalanced	1/3	2/3	1.0
Unbalanced	2/3	1/3	1.0
Unbalanced	0.1	0.9	0.25
Unbalanced	0.9	0.1	0.25
Unbalanced	0.1	0.9	0.5
Unbalanced	0.9	0.1	0.5
Unbalanced	0.1	0.9	1.0
Unbalanced	0.9	0.1	1.0

#### Amount of data

All of the above cases can produce violations of the testable implications of the underlying models depicted in Figs. 3 and 4. However, the actual causal false-positive risk will depend also on sample size. For large sample size *n*, the consistency of Bayesian posterior distributions – see Doob [38], Ghosal and Van der Vaart [39] or Ghosal and Ghosh [40] – will guarantee that the true parameter value *α*,*β* and *γ* is identified, as long as no testable implications of the underlying structural causal model are violated. However, for increasing sample size violations of the testable implications will occur with probability decreasing to zero because of the aforementioned posterior consistency. Thus, it is reasonable to study the resulting causal false-positive risk for sample sizes ranging from *n*=10 to *n*=100 samples for each observable variable. As often is the case in medical research, attaining large sample sizes may be prohibitively difficult due to cost or time constraints (e.g. the study of rare diseases), and therefore causal inference from purely observational data is even more mandated as in other scientific areas. In the simulation study, balanced sample sizes are investigated because often, there is data available for each variable in the DAG for each study participant. However, the situation of missing data could additionally induce bias and increase the false-positive risk, but this analysis is outside the scope of the current paper.

#### Simulation design

Throughout all simulations Gaussian linear models are assumed and it is further supposed that data have been standardized to z-scores, that is, *X*,*Y*,*C*,*C* in Figs. 3 to 4 are distributed as $\mathcal {N}(0,1)$, standard normal. For example, *X* could measure the methotrexate dose in milligrams a patient with rheumatoid arthritis (RA) administers weekly, *Y* could be the corresponding rheumatoid factor in a blood sample taken at a fixed time after treatment, *C* could be the physical activity measured in minutes and *W* could be additional cryotherapy interventions (also measured in minutes). Standardizing all of these units yields $X,Y,C,W \sim \mathcal {N}(0,1)$, and while this may be a somewhat simplifying assumption it allows to separate between the influence of different marginal distributions for the variables corresponding to a node in the DAG and the effect of violations of testable implications, the magnitude of the underlying structural coefficients and the influence of sample size. It is well known that distributional differences can severely influence traditional type I or II error rates (and even Bayesian error rates) in statistical hypothesis testing [22, 41], so using z-scores is a reasonable assumption. Also, we assume standard normal error terms *U*_*X*_,*U*_*Y*_,*U*_*C*_,*U*_*M*_ in the structural equations.

In the simulation study, Monte Carlo estimates for the causal false-positive risk under each of the settings outlined above are produced as the number of simulations with at least one violation of a testable implication of the underlying model divided by the total number of simulations for the model. For each model, confounders and colliders, *n*=10000 Monte Carlo simulations are run for sample sizes ranging from *n*=10 to *n*=100 for each of the structural coefficient settings for *α*,*β*,*γ*,*δ*,*ε* detailed above. Convergence to the posterior distribution in a Bayesian analysis is important, and the latter was checked via the Gelman-Rubin shrink factor [36, 42] and the effective sample size [43]. In all simulations which were conducted, no model fit via the Hamiltonian Monte Carlo sampler Stan [36, 44] showed problems in converging to the posterior based on these two convergence diagnostics^2^.

A testable implication was defined to be violated when the Bayes factor in favour (or against) the corresponding hypothesis (depending on the testable implication) passes the threshold 1. For example, the testable implication *β*_*YX*|*C*_=0 in Eq. (15) is violated when the Bayes factor *BF*_01_ in favour of *H*_0_:*β*_*YX*|*C*_=0 and against *H*_1_:*β*_*YX*|*C*_≠0 is smaller than 1. Thus, the perspective of a hard-nosed sceptic is taken who posits his structural causal model as a possible explanation of the underlying data-generating process, but who will readily modify the model when contradictions to the testable implications of the posited model arise. In the Bayesian regression models, a non-informative standard normal prior is assigned to each regression coefficient *β*, which amounts to frequentist ridge regression, compare Hastie, Tibshirani and Wainwright [45]. Using such priors is equivalent to frequentist ridge-regression, and also controls for the rate of false-positive results, compare Gelman et al. [37].

Next to the Monte Carlo estimates for the causal false-positive risk, estimates for the average causal effect *ACE*(*X*,*Y*) of *X* on *Y* as well as the direct and indirect effect *DE*(*X*,*Y*) and *IE*(*X*,*Y*) are produced. The estimands depend on possible violations of the corresponding testable implications of each model and the detailed modifications of the true DAG in each case. When no testable implications are violated by means of a hypothesis test, the correct estimands are used. The resulting Monte Carlo estimates are thus biased and provide insights how reliable causal effect estimates are given the risk of incorrectly modifying the true causal model.

## Discussion

### Confounders

**Fig. 7:**
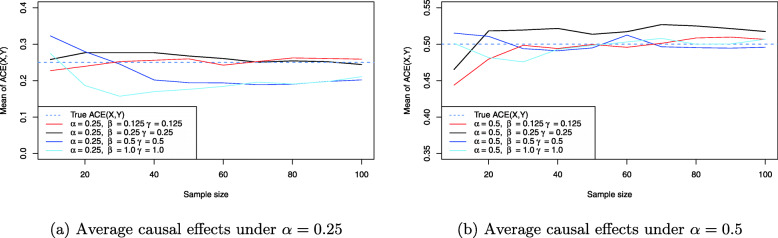
ACE(X,Y) estimates for the balanced confounder situation in Fig. 3 based on the simulation results

Figure 7 shows the resulting average causal effect estimates based on the simulations under *α*=0.25 and *α*=0.5. Importantly, from the earlier analysis it follows that the average causal effect ACE(X,Y) and the direct effect DE(X,Y) are always equal, and also IE(X,Y) is always zero, no matter whether a testable implication is violated or not. Thus, as a consquence a separate analysis for the indirect effect IE(X,Y) is not necessary, compare Lemma 1 and Table 1 in the Supplementary file, as well as Eq. (11), and all results for ACE(X,Y) apply also to DE(X,Y), compare Eqs. (9) and (11).

**Fig. 8:**
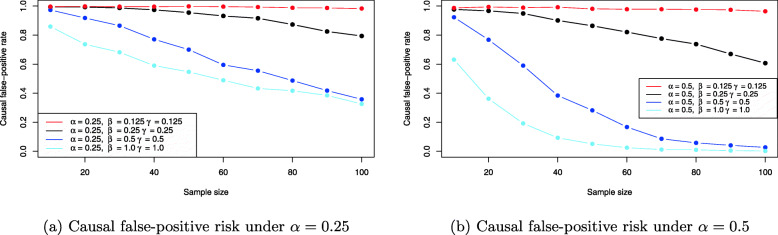
Causal false-positive risk estimates for the balanced confounder situation in Fig. 3 based on the simulation results

From Fig. 7a it can be observed that the stronger the effect of balanced confounding, the larger the induced bias on the average causal effect ACE(X,Y). For example, for *β*=*γ*=0.125 (red line) the average causal effect is reliably estimated for even moderate sample size. Shifting to balanced confounding *β*=*γ*=0.25, the situation is similar, but increasing the balanced confounding to strong or very strong influence (blue and skyblue lines) shows that the average causal effect ACE(X,Y) becomes biased more and more. For *β*=*γ*=1 even *n*=100 samples for all three observable variables *X*,*Y* and *C* do not suffice to produce a reliable estimate: The Monte Carlo estimate for ACE(X,Y) in this case yields ACE(X,Y)≈0.21 which equals about 20% bias compared to the true ACE(X,Y)=*α*=0.25. As a comparison, Fig. 7b shows the same situation for *α*=0.5, where the treatment *X* has a much stronger causal effect on the outcome *Y*. The bias introduced by confounding then reduces substantially for identical values of *β* and *γ* under balanced confounding. However, it is obvious that when *β* and *γ* increase together with *α*, the situation of Fig. 7a will be recovered again. Figure 8 shows the resulting causal false-positive risk of the confounder simulations. Figure 8a shows the results under *α*=0.25, and these show that the weaker the effect of confounding, the larger the resulting false-positive rate. Thus, when *β*=*γ*=0.125 (red line), nearly all analyses yield an error and reject the true model. Increasing the effect of balanced confounding over *β*=*γ*=0.25 to *β*=*γ*=1.0 shows that the causal false-positive risk decreases accordingly. This is to be expected because the weaker any relationship between two observable variables in the DAG in Fig. 3, the more likely it is to commit a type I error and reject the true data-generating model. However, comparison of Figs. 7a and 8a shows that a smaller false-positive rate does not necessarily imply that the effect estimates for the average causal effect ACE(X,Y) are less biased. The situation of very strong confounding *β*=*γ*=1 yields the largest bias in Fig. 7a but the smallest false-positive rate in Fig. 8a as shown by the skyblue solid line. The reason is that strong confounding (that is, the larger magnitude of the structural coefficients *β* and *γ*) may allow to identify the confounder model in Fig. 3 more reliably, but the average causal effect estimate ACE(X,Y) still is biased more than in any other setting, because whenever the strong confounding situation is *not* detected the difference between the true ACE(X,Y)=*β*_*YX*|*C*_ and the falsely calculated ACE(X,Y) (either ACE(X,Y)=0 or ACE(X,Y)=*β*_*YX*_, compare the section on testable implications and graph modifications) is substantial. As a consequence, these (rare) cases where strong or very strong confounding is not discovered bias the resulting ACE(X,Y) estimate which in turn shrinks the Monte Carlo estimate for ACE(X,Y) shown in Fig. 7a.

Figure 7b confirms this observation: Although the false-positive rate for larger *α*=0.5 decreases even more quickly for identical sample size, this does not allow to infer that the corresponding ACE(X,Y) estimates are less biased, compare Fig. 7b.

**Fig. 9:**
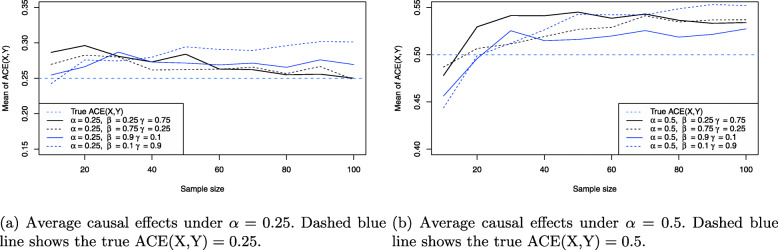
ACE(X,Y) estimates for the unbalanced confounder situation in Fig. 3 based on the simulation results

Turning to the situation of unbalanced confounding, Figure 9 shows the resulting ACE(X,Y) estimates under *α*=0.25 and *α*=0.5. Now, Fig. 9a shows that unbalanced confounding can also bias the resulting average causal effect estimates. For example, while for *β*=1/3,*γ*=0.75 (solid black line) or *β*=0.75,*γ*=1/3 (dashed black line) the resulting ACE(X,Y) estimates become approximately unbiased for a sample size of 100 samples for each observable variable *X*,*Y* and *C*, stronger imbalance can yield stronger bias: For *β*=0.1,*γ*=0.9 (dashed blue line) the resulting ACE(X,Y) Monte Carlo estimate for this sample size is ACE(X,Y)=0.30, which is 20% off the true ACE(X,Y)=0.25 shown as the horizontal dashed blue line. The situation for *β*=0.9,*γ*=0.1 is similar although not as strongly biased as for the setting *β*=0.1,*γ*=0.9. This difference can be attributed to the influence of specific values of *β* and *γ* on the occurrence of any violations of the testable implications of the confounder model as analyzed in detail in the section on testable implications and graph modifications (compare Supplementaryfile, in particular Table 1). Conceptually, changing *β* and *γ* changes the distribution of falsely applied DAG modifications in the long-term, which in turn influences the bias on the ACE(X,Y) estimates. The same phenomenon is observed in Fig. 9b where again *β*=0.1,*γ*=0.9 (dashed blue line) yields the largest bias, although the bias now is considerably less severe because *α*=0.5. This can also be explained by the fact that the worst case scenario in Fig. 3 corresponds to questioning the arrow *X*→*Y*, that is, questioning the direct effect of treatment *X* on outcome *Y*. Whenever this testable implication is violated, the induced bias is extreme, which will happen with less probability when *α* grows. As a consequence, the bias in Fig. 9b is considerably smaller than in Fig. 9a as was already the case in Fig. 7a and b.

**Fig. 10:**
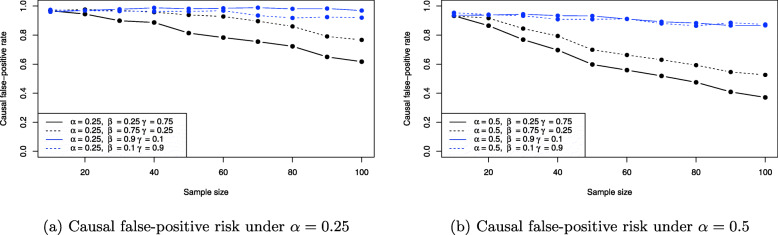
Causal false-positive risk estimates for the unbalanced confounder situation in Fig. 3 based on the simulation results

The causal false-positive risk under unbalanced confounding are shown in Fig. 10a and b. Interestingly, in the unbalanced setting a smaller false-positive rate seems to be associated with smaller bias in the corresponding ACE(X,Y) estimates: The black lines which correspond to more balanced situations yield smaller false-positive rates, and also less bias when *α*=0.25. However, switching to *α*=0.5 shows that it is in general not possible to relate the bias and false-positive risk in such a way, as e.g. the solid blue line in Fig. 9b shows that less bias is induced by *β*=0.9,*γ*=0.1 than for the more balanced settings, although the corresponding false-positive rate in Fig. 10b is much higher.

### Colliders

**Fig. 11:**
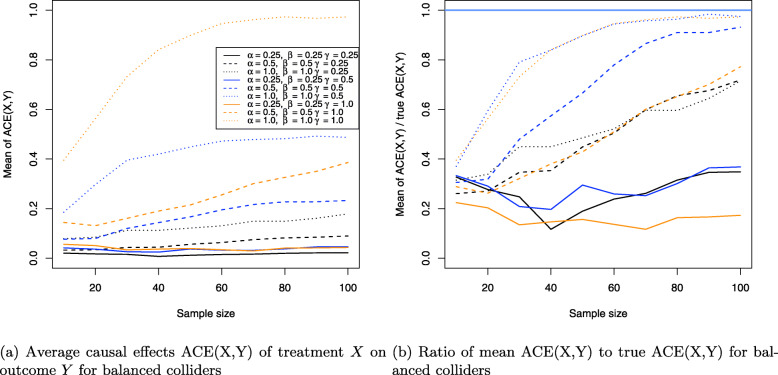
Average causal effects of treatment *X* on outcome *Y* for balanced colliders

**Fig. 12:**
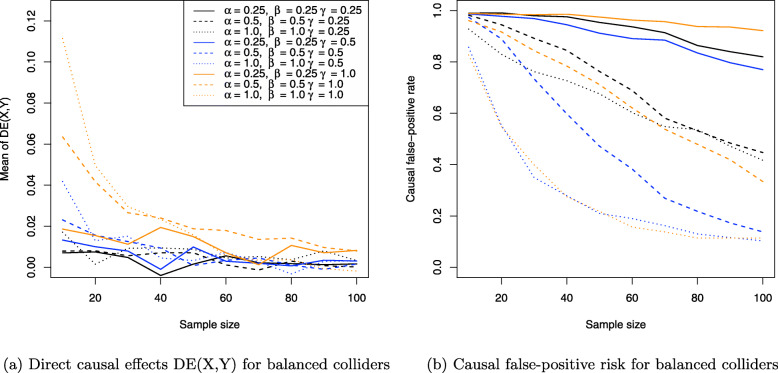
Direct causal effects of treatment *X* on outcome *Y* and the causal false-positive risk for balanced colliders

**Fig. 13:**
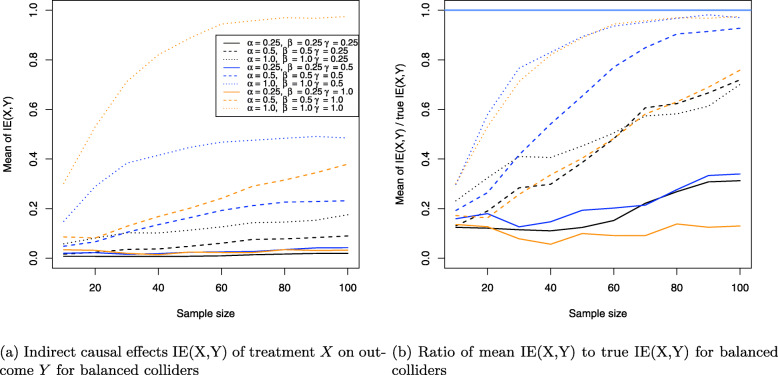
Indirect causal effects of treatment *X* on outcome *Y* for balanced colliders

The results for the collider setting in Fig. 4 are shown in Figs. 11, 12 and 13. Figure 11a shows the Monte Carlo estimates for the average causal effect ACE(X,Y) of treatment *X* on outcome *Y* and evidently, there is strong bias in almost every simulation setting. This is also reflected in Fig. 11b which shows the ratio of the Monte Carlo estimates and true ACE(X,Y) for each setting. Consequently, the ACE(X,Y) is estimated nearly unbiased when the corresponding line comes close to the horizontal blue line at the ratio 1, which holds only for the three settings where two coefficients have magnitude of 1 (dotted yellow and blue lines) or all three coefficients magnitude of 0.5 (dashed blue line), with the latter setting exhibiting a slightly stronger bias. All other settings suffer from much stronger bias.

Figure 12a and b show the simulation results for the direct causal effects and causal false-positive risk for balanced colliders. Based on Fig. 12a, the true direct effect DE(X,Y)=0 is identified correctly for large enough sample size in any setting, although larger structural coefficients imply larger bias which again shows that a larger magnitude of structural coefficients may not have a direct relationship to the induced bias. While it is often perceived that such stronger relationships are easier to identify, the bias on the causal effects may be much higher than for more moderate magnitudes. That is, the induced bias is not (negatively) proportional to the magnitude of structural coefficients. This is reflected for example in the green dotted and dashed lines in Fig. 12a which correspond to the largest structural coefficients but also induce the strongest bias on DE(X,Y). Inspecting the causal false-positive risk in Fig. 12b shows that the magnitude of structural coefficients *α*,*β* and *γ*, however, relates directly to the associated false-positive risk. The dotted yellow and blue lines and the dashed blue line correspond to the largest structural coefficients and imply the smallest false-positive risk. The probability to reject the true DAG for these settings is thus smallest among all settings. The weaker the relationship between the observable variables, the higher the false-positive risk. For balanced colliders, the causal false-positive risk is thus a direct indicator of the presence (not the magnitude) of bias on the average causal, direct and indirect causal effects.

Figure 13a and b confirm this phenomenon, as the situation for the indirect causal effects is similar to the situation for the average causal effects in Fig. 11a and b.

**Fig. 14:**
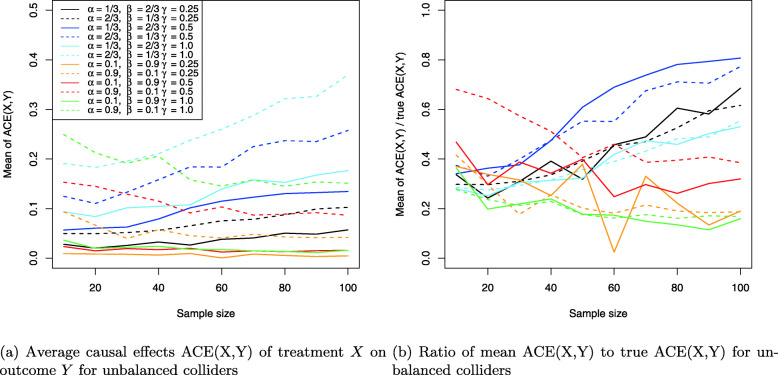
Average causal effects of treatment *X* on outcome *Y* for unbalanced colliders

Turning to the situation of unbalanced colliders, two phenomena can be observed. First, all settings with *α*=0.1 (solid yellow, orange and red lines) yield the strongest bias as shown by the ratio of Monte Carlo estimates for ACE(X,Y) and true ACE(X,Y) in Fig. 14b, compare also Fig. 14a. Note that this is not simply because for *α*=0.1, the probability of deleting the arrow *X*→*C* is largest in Fig. 4, which in turn implies that ACE(X,Y) vanishes entirely. In fact, from Fig. 14b it becomes clear that even for *α*=0.9,*β*=0.1,*γ*=1.0 (green dashed line), the induced bias on ACE(X,Y) is comparable and larger than in all other settings except for the setting *α*=0.1,*β*=0.9,*γ*=1.0 (solid green line). Thus, imbalance can function as a catalyst for the induced bias on the average causal effect in collider settings. Second, Fig. 14b shows that for the most balanced settings (blue and black solid and dashed lines) the bias is weakest among all settings.

**Fig. 15:**
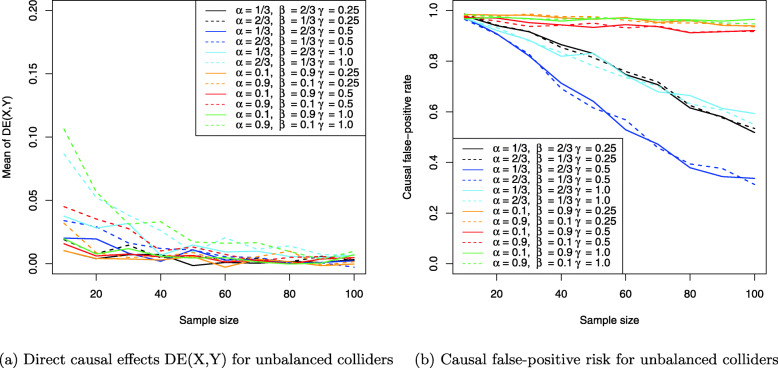
Direct causal effects of treatment *X* on outcome *Y* and the causal false-positive risk for unbalanced colliders

Shifting to the direct causal effects of unbalanced colliders, Fig. 15a and b shows the same phenomenon as for balanced colliders. The direct causal effect DE(X,Y) is revealed correctly but larger imbalance implies larger bias here, too. Also, Fig. 15b shows that the larger the imbalance, the larger the causal false-positive risk: The dashed and solid blue lines correspond to the most balanced setting, yielding the smallest error rates. The dashed and solid black lines correspond to the second-most balanced setting, yielding slightly larger error rates. The same holds in turn for the skyblue dashed and solid lines, and the green, orange and red lines are the most imbalanced collider settings, being associated with the largest probability to incorrectly reject the true causal model.

**Fig. 16:**
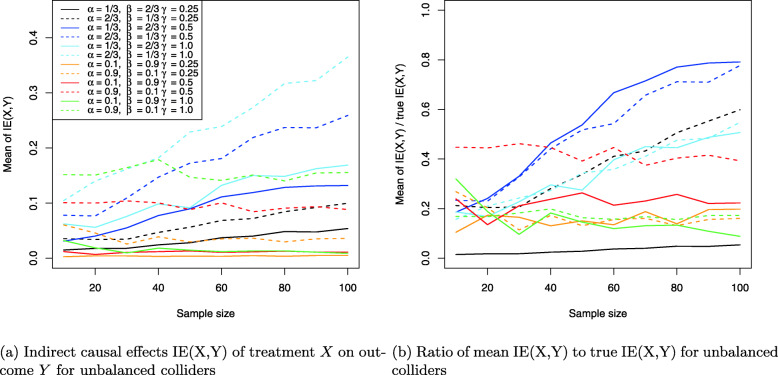
Indirect causal effects of treatment *X* on outcome *Y* for unbalanced colliders

Figure 16a and b confirm the phenomena observed already in Fig. 14a and b for the average causal effects ACE(X,Y) for the indirect causal effects IE(X,Y).

## Conclusions

Causal inference has become an important methodology in medical research, in particular, when a randomized controlled trial is not possible. Even when randomization is possible, estimation of direct and indirect effects is crucial in the presence of confounders or colliders. Qualitative causal assumptions – often expressed as a directed acyclic graph (DAG) – and experimental or even non-experimental data can yield quantitative causal inferences in such settings. However, although the identification and calculation of structural coefficients in such models has received much attention, a key premise for valid causal inference is that conclusions are drawn based on the true data-generating model.

By now, it remained widely unknown how large the probability is to reject the true structural causal model after observational data from it is recorded. The latter probability – the causal false-positive risk – is crucial, since rejecting the true causal model can lead to bias in the estimation of structural coefficients and causal effects, thus producing false causal conclusions. In this paper, the building blocks of structural causal models were studied regarding their associated causal false-positive risk. A simulation study was carried out which investigated the probability that elementary causal structures such as confounders and colliders in a DAG are misclassified. Therefore, the testable implications of the DAG were analyzed and the corresponding modifications derived based on available theory of directed acyclic graphs.

Results showed that the false-positive risk of rejecting a true but simple causal model like confounders or colliders is substantial. The probability to falsely reject even a simple causal model turned out to be substantial in all simulations. Importantly, estimation of average causal effects can become biased quickly if a true model is rejected (for the case of balanced confounders, see Fig. 7, for the case of balanced colliders, see Fig. 11). While this already holds in the balanced settings, introducing imbalance – which is more realistic in practice – even works as a catalyst on the induced biases (compare the analysis in the previous section). For direct and indirect causal effects, the same applies: For balanced confounders, the direct effect and average causal effect coincide even if the true causal model is falsely rejected, and thus the bias on DE(X,Y) equals the bias on ACE(X,Y). Adding imbalance to the confounding mechanism, the bias can increase (compare Fig. 9a). For the direct and indirect causal effects in the collider model, substantial bias was strongly correlated with a high false-positive risk (compare Figs. 12 and 13), as was already the case for the average causal effects (compare Fig. 11). Additional imbalance here also functioned as a catalyst which drove up the induced bias as shown in Figs. 14, 15 and 16. Importantly, a high false-positive risk is strongly correlated with large bias on the causal effect estimates. The latter fact is natural since rejecting the true structural causal model often (but not always) amounts to an incorrect modification based on the observed violation of a testable implication of the model. The according change in the estimands for the average, direct and indirect causal effects can thus induce bias on the resulting estimates. This is different to the situation of a confounder, where a larger false-positive risk could not be equated with a larger induced bias.

However, there are also limitations of the results presented herein. First, linear Markovian models are not appropriate in every situation, and semi-Markovian models may be more realistic in a variety of cases, allowing for correlated error terms among the observable variables. Furthermore, the causal false-positive risk decreases to zero whenever the association between variables is strong enough. Still, in the biomedical and cognitive sciences small to medium effect sizes are the norm rather the exception – compare Aarts et al. [46]. Third, Bayesian analysis of the testable implications is associated with the priors on the regression coefficients. However, as the same weakly-informative priors were chosen in all situations, results are comparable [11, 36]. These priors also control for false-positive results, compare Gelman et al. [37]. Also, for increasing sample size the influence of the prior becomes negligible, and the results showed that even for sample sizes of 100 observations per observable variable in the causal model, the false-positive risk often stays above 50%, yielding a substantial probability to reject the true causal model. As a consequence, the results presented in this paper can also be perceived as a kind of causal power analysis for simple confounder and collider settings, which shows that to reliable identify the true causal model, sample sizes of 100 are not enough in observational studies. This is insofar important, as obtaining large sample sizes often is difficult in medical research, e.g. in the study of rare diseases.

Fourth, next to the influence of the prior distribution in a Bayesian analysis the choice of the statistical evidence measure to test a hypothesis in form of a testable implication is relevant. As discussed in the section about the statistical analysis of the testable implications, the Bayes factor which was used in the simulations is one of the most widely established approaches to hypothesis testing in medical research, but there are alternatives. Future research should study whether the same conclusions can be drawn regarding the causal false-positive risk and the induced bias when a different approach to Bayesian hypothesis testing is taken. Importantly, the results obtained here hold only when using the Bayes factor for testing the testable implications.

An interesting extension of the current work would be to study other important causal models regarding their associated causal false-positive risk, such as mediators or confounded mediators. However, obtaining theoretical results such as Lemmas 1 or 2 becomes necessarily more complicated in more complex structural causal models, because the number of testable implications quickly becomes large.

In sum, while the identification of structural coefficients and testable implications in causal inference have been studied rigorously in the literature, the results of this paper show that causal inference also must develop new concepts for controlling the causal false-positive risk, as the latter often is strongly correlated with the induced bias on the estimated average, direct and indirect causal effects (of a treatment *X* on outcome *Y*). Although a high false-positive risk cannot be equated with a substantial bias by theoretical means by now, the latter fact calls for the development of new and more elaborate risk measures for wrongly rejecting the true causal model in causal inference.

## Supplementary Information

**Additional file 1** Supplementary materials.

## Data Availability

All results and figures can be reproduced by the replication script available at the Open Science Foundation at https://osf.io/fmqjz/?view_only=719c493588d5406394c8f393d3b16249.

## Abbreviations

CSM: Structural causal model
RCT: Randomized controlled trial
DAG: Directed acyclic graph
RA: Rheumatoid arthritis
PT: Physical therapy
ACE: Average causal effect
DE: Direct effect
IE: Indirect effect

## Footnotes

1 Note, however, that the estimate for a causal effect must not necessarily be biased. There may exist causal effects which are identical under different structural causal models. Still, often this is not the case so some bias will be induced by shifting to a wrong causal model.

2 Compare the provided replication scripts. All results and figures can be reproduced via the replication script at the Open Science Foundation under https://osf.io/fmqjz/?view_only=719c493588d5406394c8f393d3b16249.

## References

[1] Pearl J (2009). Causality: Models, Reasoning, and Inference, Second Edition.

[2] Pearl J, Glymour M, Jewell NP (2016). Causal Inference in Statistics: A Primer.

[3] Fisher RA (1935). The Design of Experiments, 1st ed.

[4] VanderWeele TJ (2015). Explanation in Causal Inference: Methods for Mediation and Interaction.

[5] Walker M (2017). Why We Sleep: The New Science of Sleep and Dreams.

[6] Dawid AP (2015). Statistical Causality from a Decision-Theoretic Perspective. Ann Rev Stat Appl.

[7] VanderWeele TJ. Mediation Analysis: A Practitioner’s Guide. Ann Rev Inc. 2016. 10.1146/annurev-publhealth-032315-021402. 10.1146/annurev-publhealth-032315-021402 26653405

[8] Pearl J, MacKenzie D (2018). The Book of Why.

[9] Verma T, Pearl J (1988). Causal networks: Semantics and expressiveness. Proceedings of the Fourth Workshop on Uncertainty in Artificial Intelligence.

[10] Kelter R. Analysis of Bayesian posterior significance and effect size indices for the two-sample t-test to support reproducible medical research. BMC Med Res Methodol. 2020; 20(88). 10.1186/s12874-020-00968-2. 10.1186/s12874-020-00968-2 PMC7178740 32321438

[11] Kelter R. Bayesian alternatives to null hypothesis significance testing in biomedical research: a non-technical introduction to Bayesian inference with JASP. BMC Med Res Methodol. 2020; 20(1). 10.1186/s12874-020-00980-6. 10.1186/s12874-020-00980-6 PMC7275319 32503439

[12] Kelter R (2020). Bayesian survival analysis in STAN for improved measuring of uncertainty in parameter estimates. Meas Interdiscip Res Perspect.

[13] Wagenmakers E-J, Morey RD, Lee MD (2016). Bayesian Benefits for the Pragmatic Researcher. Curr Dir Psychol Sci.

[14] Lauritzen SL, Dawid AP, Larsen BN, Leimer HG (1990). Independence properties of directed Markov fields. Networks.

[15] Bollen KA (1989). Structural Equations with Latent Variables.

[16] Wright S (1921). Correlation and Causation. J Agric Res.

[17] Chen B, Pearl J. Graphical Tools for Linear Structural Equation Modeling. Technical report, University of California, Los Angeles, Computer Science Department, Los Angeles. 2015.

[18] Berzuini C, Dawid P, Bernardinell L, VanderWeele TJ, Hernán MA (2012). Causality: Statistical Perspectives and Applications.

[19] Pearl J (1998). Graphs, causality, and structural equation models. Sociol Methods Res.

[20] Spirtes P, Richardson T, Meek C, Scheines R, Glymour C (1998). Using path diagrams as a structural equation modeling tool. Sociol Methods Res.

[21] Hernán M, Robins J (2020). Causal Inference: What If.

[22] Kelter R. Bayesian and frequentist testing for differences between two groups with parametric and nonparametric two-sample tests. WIREs Comput Stat. 2021;13(6). 10.1002/wics.1523.

[23] van Erp S, Oberski DL, Mulder J (2019). Shrinkage priors for Bayesian penalized regression. J Math Psychol.

[24] Robert CP (2007). The Bayesian Choice, 2nd ed.

[25] Kruschke JK (2014). Doing Bayesian Data Analysis: A Tutorial with R, JAGS, and Stan, 2nd ed.

[26] Berger JO (1985). Statistical Decision Theory and Bayesian Analysis.

[27] Schervish MJ (1995). Theory of Statistics.

[28] Kelter R (2021). How to Choose between Different Bayesian Posterior Indices for Hypothesis Testing in Practice. Multivar Behav Res.

[29] Makowski D, Ben-Shachar MS, Chen SHA, Lüdecke D (2019). Indices of Effect Existence and Significance in the Bayesian Framework. Front Psychol.

[30] Linde M, Tendeiro J, Selker R, Wagenmakers E-J, van Ravenzwaaij D. Decisions About Equivalence: A Comparison of TOST, HDI-ROPE, and the Bayes Factor. psyarxiv preprint. 2020. https://psyarxiv.com/bh8vu. 10.1037/met0000402 34735173

[31] Kelter R. Bayesian Hodges-Lehmann tests for statistical equivalence in the two-sample setting: Power analysis, type I error rates and equivalence boundary selection in biomedical research. BMC Med Res Methodol. 2021; 21(1). 10.1186/s12874-021-01341-7. 10.1186/s12874-021-01341-7 PMC8369333 34404344

[32] Kelter R. fbst: An R package for the Full Bayesian Significance Test for testing a sharp null hypothesis against its alternative via the e-value. Behav Res Methods. 2021; (in press). 10.3758/s13428-021-01613-6. 10.3758/s13428-021-01613-6 PMC9170675 34471963

[33] Ly A, Verhagen J, Wagenmakers E-J (2016). An evaluation of alternative methods for testing hypotheses, from the perspective of Harold Jeffreys. J Math Psychol.

[34] Berger JO, Boukai B, Wang Y (1997). Unified Frequentist and Bayesian Testing of a Precise Hypothesis. Stat Sci.

[35] Sellke T, Bayarri MJ, Berger JO (2001). Calibration of p values for testing precise null hypotheses. Am Stat.

[36] Goodrich B, Gabry J, Ali I, Brilleman S. rstanarm: Bayesian applied regression modeling via Stan. R package version 2.19.3. 2020. https://mc-stan.org/rstanarm/articles/priors.html.

[37] Gelman A, Hill J, Yajima M (2012). Why We (Usually) Don’t Have to Worry About Multiple Comparisons. Journal of Research on Educational Effectiveness.

[38] Doob JL (1949). Le Calcul des Probabilités et ses Applications. Colloques Internationaux Du Centre National de La Recherche Scientifique, No. 13. Centre National de la Recherche Scientifique, Paris.

[39] Ghosal S, van der Vaart A. Fundamentals of Nonparametric Bayesian Inference; 2017. 10.1017/9781139029834.

[40] Ghosal S, Ghosh JK, van der Vaart AW (2000). Convergence rates of posterior distributions. Ann Stat.

[41] Kelter R. Analysis of type I and II error rates of Bayesian and frequentist parametric and nonparametric two-sample hypothesis tests under preliminary assessment of normality. Comput Stat. 2020; (in press). 10.1007/s00180-020-01034-7.

[42] Gelman A, Rubin DB (1992). Inference from Iterative Simulation Using Multiple Sequences. Stat Sci.

[43] Robert C, Casella G (2004). Monte Carlo Statistical Methods.

[44] Gelman A, Lee D, Guo J (2015). Stan: A Probabilistic Programming Language for Bayesian Inference. J Educ Behav Stat.

[45] Hastie T, Tibshirani R, Wainwright M (2015). Statistical Learning with Sparsity : the Lasso and Generalizations, 1st ed.

[46] Aarts AA, Anderson JE, Anderson CJ, Attridge PR, Attwood A, Axt J, Babel M, Bahník Š, Baranski E, Barnett-Cowan M, Bartmess E, Beer J, Bell R, Bentley H, Beyan L, Binion G, Borsboom D, Bosch A, Bosco FA, Bowman SD, Brandt MJ, Braswell E (2015). Estimating the reproducibility of psychological science. Science.

