# Supplementary material for: Bayesian identification of structural coefficients in causal models and the causal false-positive risk of confounders and colliders in linear Markovian models
Source: BMC Med Res Methodol. 2022 Feb 27;22:58. doi: 10.1186/s12874-021-01473-w (PMC8883695; doi:10.1186/s12874-021-01473-w)
Supplement: Supplementary file 1 — Additional file 1 Supplementary materials. [file 12874_2021_1473_MOESM1_ESM.pdf]

Supplementary file to *Bayesian identification of structural coefficients in causal models and the causal false-positive risk of confounders and colliders in linear Markovian models*

Proof of Lemma 1

Based on the backdoor-criterion and single-door criterion in Theorem 3 and 4, Table 1 provides the resulting estimands for ACE(X,Y), DE(X,Y) and IE(X,Y) for the confounder situation shown in Fig. 3 based on which the induced bias can be calculated from Equations (9), (11) and (12).

	New estimands		
Violation	ACE(X,Y)	DE(X,Y)	IE(X,Y)
$k = 1$ testable implications violated			
$\alpha = 0$	0	0	0
$\beta = 0$	β_{YX}	β_{YX}	0
$\gamma = 0$	β_{YX}	β_{YX}	0
$k = 2$ testable implications violated			
$\alpha = 0$, $\beta = 0$	0	0	0
$\alpha = 0$, $\gamma = 0$	0	0	0
$\beta = 0, \gamma = 0$	β_{YX}	β_{YX}	0
$k = 3$ testable implications violated			
$\alpha = 0$, $\beta = 0$, $\gamma = 0$	0	0	0

Table 1: Resulting estimands for ACE(X,Y), DE(X,Y) and IE(X,Y) and induced bias for the confounder model in Fig. 3

Proof of Lemma 2 Assume the true structural causal model is given by the directed acyclic graph G in Figure 4 with testable implications given in Equation (15). The induced bias on ACE(X,Y), DE(X,Y), IE(X,Y) for each possible violation of one or multiple testable implications (each case of which contributes to the causal-false positive risk) can be derived from the resulting graph modifications and the single-door and back-door criteria in Theorems 3 and 4, and are given in Table 2 and 3 under Assumption 1.

							New estimands		
Violation	α	β	γ	ξ	ε	δ	DE(X,Y)	IE(X,Y)	ACE(X,Y)
$k=0$ testable implications violated									
	$\neq 0$	$\neq 0$	$\neq 0$	0	0	0	0	$\beta_{CX} \cdot \beta_{YC}$	$\beta_{CX} \cdot \beta_{YC}$
$k=1$ testable implications violated									
	0	$\neq 0$	$\neq 0$	0	0	0	0	0	0
	$\neq 0$	0	$\neq 0$	0	0	0	0	0	0
	$\neq 0$	$\neq 0$	0	0	0	0	0	0	0
	$\neq 0$	$\neq 0$	$\neq 0$	$\neq 0$	0	0	0	$\beta_{CX\|W} \cdot \beta_{YC}$	$\beta_{CX\|W} \cdot \beta_{YC}$
	$\neq 0$	$\neq 0$	$\neq 0$	0	$\neq 0$	0	0	$\beta_{YC\|W} \cdot \beta_{CX}$	$\beta_{YC\|W} \cdot \beta_{CX}$
	$\neq 0$	$\neq 0$	$\neq 0$	0	0	$\neq 0$	$\beta_{YX\|C}$	$\beta_{YC\|X} \cdot \beta_{CX}$	$\beta_{YX\|C} + \beta_{YC\|X} \cdot \beta_{CX}$
$k=2$ testable implications violated									
	0	0	$\neq 0$	0	0	0	0	0	0
	0	$\neq 0$	0	0	0	0	0	0	0
	0	$\neq 0$	$\neq 0$	$\neq 0$	0	0	0	0	0
	0	$\neq 0$	$\neq 0$	0	$\neq 0$	0	0	0	0
	0	$\neq 0$	$\neq 0$	0	0	$\neq 0$	β_{YX}	0	β_{YX}
	$\neq 0$	0	0	0	0	0	0	0	0
	$\neq 0$	0	$\neq 0$	$\neq 0$	0	0	0	$\beta_{CX} \cdot \beta_{YC}$	$\beta_{CX} \cdot \beta_{YC}$
	$\neq 0$	0	$\neq 0$	0	$\neq 0$	0	0	$\beta_{CX} \cdot \beta_{YC}$	$\beta_{CX} \cdot \beta_{YC}$
	$\neq 0$	0	$\neq 0$	0	0	$\neq 0$	$\beta_{YX\|C}$	$\beta_{CX} \cdot \beta_{YC\|X}$	$\beta_{CX} \cdot \beta_{YC\|X}$
	$\neq 0$	$\neq 0$	0	$\neq 0$	0	0	0	0	0
	$\neq 0$	$\neq 0$	0	0	$\neq 0$	0	0	0	0
	$\neq 0$	$\neq 0$	0	0	0	$\neq 0$	β_{YX}	0	β_{YX}
	$\neq 0$	$\neq 0$	$\neq 0$	$\neq 0$	$\neq 0$	0	0	$\beta_{CX\|W} \cdot \beta_{YC\|W}$	$\beta_{CX\|W} \cdot \beta_{YC\|W}$
	$\neq 0$	$\neq 0$	$\neq 0$	$\neq 0$	0	$\neq 0$	$\beta_{YX\|C}$	$\beta_{CX\|W} \cdot \beta_{YC\|X}$	$\beta_{CX\|W} \cdot \beta_{YC\|X}$
	$\neq 0$	$\neq 0$	$\neq 0$	0	$\neq 0$	$\neq 0$	$\beta_{YX\|C,W}$	$\beta_{CX} \cdot \beta_{YC\|X,W}$	$\beta_{CX} \cdot \beta_{YC\|X,W}$
$k=3$ testable implications violated									
	0	0	0	0	0	0	0	0	0
	0	0	$\neq 0$	$\neq 0$	0	0	0	0	0
	0	0	$\neq 0$	0	$\neq 0$	0	0	0	0
	0	0	$\neq 0$	0	0	$\neq 0$	β_{YX}	0	β_{YX}
	$\neq 0$	0	0	$\neq 0$	0	0	0	0	0
	$\neq 0$	0	0	0	$\neq 0$	0	0	0	0
	$\neq 0$	0	0	0	0	$\neq 0$	β_{YX}	0	β_{YX}
	$\neq 0$	$\neq 0$	0	$\neq 0$	$\neq 0$	0	0	0	0
	$\neq 0$	$\neq 0$	0	$\neq 0$	0	$\neq 0$	β_{YX}	0	β_{YX}
	$\neq 0$	$\neq 0$	$\neq 0$	$\neq 0$	$\neq 0$	$\neq 0$	$\beta_{YX\|W,C}$	$\beta_{YX\|W} - \beta_{YX\|W,C}$	$\beta_{YX\|W}$
	0	$\neq 0$	0	$\neq 0$	0	0	0	0	0
	0	$\neq 0$	0	0	$\neq 0$	0	0	0	0
	0	$\neq 0$	0	0	0	$\neq 0$	β_{YX}	0	β_{YX}
	0	$\neq 0$	$\neq 0$	$\neq 0$	$\neq 0$	0	0	0	0
	0	$\neq 0$	$\neq 0$	$\neq 0$	0	$\neq 0$	$\beta_{YX\|C}$	0	$\beta_{YX\|C}$
	0	$\neq 0$	$\neq 0$	0	$\neq 0$	$\neq 0$	β_{YX}	0	β_{YX}
	$\neq 0$	0	$\neq 0$	$\neq 0$	$\neq 0$	0	0	$\beta_{CX} \cdot \beta_{YC\|W}$	$\beta_{CX} \cdot \beta_{YC\|W}$
	$\neq 0$	0	$\neq 0$	$\neq 0$	0	$\neq 0$	$\beta_{YX\|C}$	$\beta_{CX} \cdot \beta_{YC\|X}$	$\beta_{YX\|C} + \beta_{CX} \cdot \beta_{YC\|X}$
	$\neq 0$	0	$\neq 0$	0	$\neq 0$	$\neq 0$	$\beta_{YX\|C}$	$\beta_{CX} \cdot \beta_{YC\|X}$	$\beta_{YX\|C} + \beta_{CX} \cdot \beta_{YC\|X}$
	$\neq 0$	$\neq 0$	0	0	$\neq 0$	$\neq 0$	β_{YX}	0	β_{YX}

Table 2: Resulting estimands for ACE(X,Y), DE(X,Y) and IE(X,Y) and induced bias for the collider model in Fig. 4 (Part I)

Violation	α	β	γ	ξ	ε	δ	New estimands DE(X,Y)	IE(X,Y)	ACE(X,Y)
$k = 4$ testable implications violated									
	0	0	0	$\neq 0$	0	0	0	0	0
	0	0	0	0	$\neq 0$	0	0	0	0
	0	0	0	0	0	$\neq 0$	β_{YX}	0	β_{YX}
	$\neq 0$	0	0	$\neq 0$	$\neq 0$	0	0	0	0
	$\neq 0$	0	0	$\neq 0$	0	$\neq 0$	β_{YX}	0	β_{YX}
	0	$\neq 0$	0	$\neq 0$	$\neq 0$	0	0	0	0
	0	$\neq 0$	0	$\neq 0$	0	$\neq 0$	β_{YX}	0	β_{YX}
	0	$\neq 0$	$\neq 0$	$\neq 0$	$\neq 0$	$\neq 0$	$\beta_{YX\|W,C}$	0	$\beta_{YX\|W,C}$
	$\neq 0$	0	$\neq 0$	$\neq 0$	$\neq 0$	$\neq 0$	$\beta_{YX\|W,C}$	$\beta_{YX\|W} - \beta_{YX\|W,C}$	$\beta_{YX\|W}$
	$\neq 0$	$\neq 0$	0	$\neq 0$	$\neq 0$	$\neq 0$	$\beta_{YX\|W}$	0	$\beta_{YX\|W}$
	$\neq 0$	0	0	0	$\neq 0$	$\neq 0$	β_{YX}	0	β_{YX}
	0	0	$\neq 0$	0	$\neq 0$	$\neq 0$	β_{YX}	0	β_{YX}
	0	0	$\neq 0$	$\neq 0$	$\neq 0$	0	0	0	0
	0	0	$\neq 0$	$\neq 0$	0	$\neq 0$	β_{YX}	0	β_{YX}
	0	$\neq 0$	0	0	$\neq 0$	$\neq 0$	$\beta_{YX\|W}$	0	$\beta_{YX\|W}$
$k = 5$ testable implications violated									
	0	0	0	$\neq 0$	$\neq 0$	0	0	0	0
	$\neq 0$	0	0	$\neq 0$	$\neq 0$	$\neq 0$	$\beta_{YX\|W}$	0	$\beta_{YX\|W}$
	0	$\neq 0$	0	$\neq 0$	$\neq 0$	$\neq 0$	$\beta_{YX\|W}$	0	$\beta_{YX\|W}$
	0	0	$\neq 0$	$\neq 0$	$\neq 0$	$\neq 0$	$\beta_{YX\|W}$	0	$\beta_{YX\|W}$
	0	0	0	0	$\neq 0$	$\neq 0$	β_{YX}	0	β_{YX}
	0	0	0	$\neq 0$	0	$\neq 0$	β_{YX}	0	β_{YX}
$k = 6$ testable implications violated									
	0	0	0	$\neq 0$	$\neq 0$	$\neq 0$	$\beta_{YX\|W}$	0	$\beta_{YX\|W}$

Table 3: Resulting estimands for ACE(X,Y), DE(X,Y) and IE(X,Y) and induced bias for the collider model in Fig. 4 (Part II)
